# An ultrasound-driven immune-boosting molecular machine for systemic tumor suppression

**DOI:** 10.1126/sciadv.abj4796

**Published:** 2021-10-20

**Authors:** Liu Wang, Guangzhe Li, Lei Cao, Yi Dong, Yang Wang, Shisheng Wang, Yueqing Li, Xiuhan Guo, Yi Zhang, Fangfang Sun, Xuemei Du, Jiangan Su, Qing Li, Xiaojun Peng, Kun Shao, Weijie Zhao

**Affiliations:** 1 State Key Laboratory of Fine Chemicals, Department of Pharmacy, School of Chemical Engineering, Dalian University of Technology, Dalian 116024, China.; 2 State Key Laboratory of Fine Chemicals, School of Chemical Engineering, Dalian University of Technology, Dalian 116024, China.; 3 Nuclear Medicine, First Affiliated Hospital of Dalian Medical University, Dalian 116021, China.; 4 EEC Biotech Co. Ltd, Guangzhou 510070, China.

## Abstract

Exploring facile and effective therapeutic modalities for synergistically controlling primary tumor and metastasis remains a pressing clinical need. Sonodynamic therapy (SDT) offers the possibility of noninvasively eradicating local solid tumors, but lacks antimetastatic activity because of its limited ability in generating systemic antitumor effect. Here, we exploited a previously unidentified ultrasound-driven “molecular machine,” DYSP-C34 (C34 for short), with multiple attractive features, emerging from preferential tumor accumulation, potent ultrasound-triggered cytotoxicity, and intrinsic immune-boosting capacity. Driven by the ultrasound, C34 functioned not only as a tumor cell killing reagent but also as an immune booster that could potentiate robust adaptive antitumor immunity by directly stimulating dendritic cells, resulting in the eradication of the primary solid tumor along with the inhibition of metastasis. This molecular machine, C34, rendered great promise to achieve systemic treatment against cancer via unimolecule-mediated SDT.

## Teaser

The “molecular machine” C34 could suppress tumor systemically via unimolecule-mediated sono/immunotherapy.

## INTRODUCTION

Cancer therapy has evolved slowly with very rare successes (*1*). Although conventional therapies, such as radiation therapy, surgery, and chemotherapy, have improved treatment effects against well-defined primary solid tumors, curative outcomes still remain unsatisfactory especially in patients with the occurrence of metastatic tumor, which actually contributes to more than 90% of mortality in cancer patients (*2*). The reason could be a result of less accessibility of most therapeutics to microscopic tumor lesions, which occurred in secondary organs. For this case, by mounting a dynamic antitumor immune response, immunotherapy has been shown to be most beneficial in tackling the tumor cells, particularly the ones hiding in the “corner” of the body (*3*, *4*). However, the immunotherapeutic effect against solid tumors was hindered by the unamiable tumor microenvironment (*5*). Therefore, the development of a novel cancer therapeutic modality, which is able to destroy the primary solid tumor together with recognizing, suppressing, and even eliminating the residual cancer cells at the site of metastasis, has been widely considered as the ultimate goal in the battle against cancer.

Being a representative noninvasive cancer treatment, sonodynamic therapy (SDT), which was adapted from photodynamic therapy (PDT), has recently garnered substantial interests in cancer therapy, mainly because of its great advantage of deep tissue penetration (*6*, *7*). SDT uses synergetic interaction between the low-intensity ultrasound (US) and a sonosensitizer to elicit cytotoxic effects. Currently, the most accepted mechanism suggests that the US-activated sonosensitizers lead to conversion of molecular oxygen to various highly reactive oxygen species (ROS), which induces apoptosis and necrosis of tumor cells (*8*, *9*).

Notably, accumulating evidence has suggested that the SDT process could initiate, but in a very limited way, host antitumor immunity (*10*) by inducing immunogenic cell death (ICD) of dying cancer cells, which is characterized by the release of “danger” signals (*11*–*13*), thus enabling antigen-presenting cells (APCs) to trigger “vaccine-like” immune responses. Unfortunately, the extent of immune responses arising from SDT is not yet robust enough to efficiently control tumor growth and metastasis (*14*, *15*). To harness the potency of the systemic antitumor immunity, the development of sono/immunocombinational therapy has been exploited as an attractive strategy to amplify the overall therapeutic effect (*16*), mostly through the nanocarriers to co-deliver the sonosensitizers and the adjuvants or checkpoint inhibitors, of which the effectiveness in mouse models has been validated in many studies (*10*, *12*, *17*). While the importance of nanomedicine continues to be highlighted, the biodegradability and biosafety of these complicated nanosystems are always brought as the major concerns for their further clinical translation. Driven by practical clinical benefits, the motivation for exploring a “small molecular machine,” which is equipped with synchronized functions of tumor targetability, acoustic responsive activity, and immune-boosting capacity to achieve profound antitumor outcomes, has become more realistic.

Natural products are a constant source of leads for many bioactive molecules, which hold promise for being developed as new medicines (*18*). Chlorin e6 (Ce6), isolated from green plants or silkworm excrement, is a second-generation sensitizer with potent sonosensitivity (*19*). A number of studies have shown that Ce6-mediated SDT was capable of suppressing the proliferation of hepatoma cells (*20*), breast cancer cells (*21*), human lung adenocarcinoma cells (*22*), etc. However, low bioavailability, poor solubility, and tendency to aggregate in the biologic medium hinder in vivo applications of Ce6 (*23*). Many new types of chlorin-based sensitizers have been extensively exploited because of their structural flexibility for straightforward chemical modification. For example, talaporfin sodium [mono-l-aspartyl Ce6 (NPe6)] has been approved in Japan in 2003 as a commercial photosensitizer for the treatment of early-stage lung cancers (*24*). Previously, in our laboratory, a chlorophyll derivative with an identical chemical structure to Ce6 was successfully prepared from the natural spirulina powders originating in Chenghai Lake in Yunnan Province, China, which was named Chenghai chlorin (CHC) (*25*). Our early findings demonstrated that the introduction of the π-extension system to the macrocyclic structure of CHC could finely modulate its photochemical features, molecular lipophilicity, and even ROS production ability toward favorable directions in PDT (*25*, *26*). On the basis of this, we suspected that the π-extension system might be able to improve sonochemical properties, SDT effect, and other molecular properties of CHC as well.

**Fig. 1.**
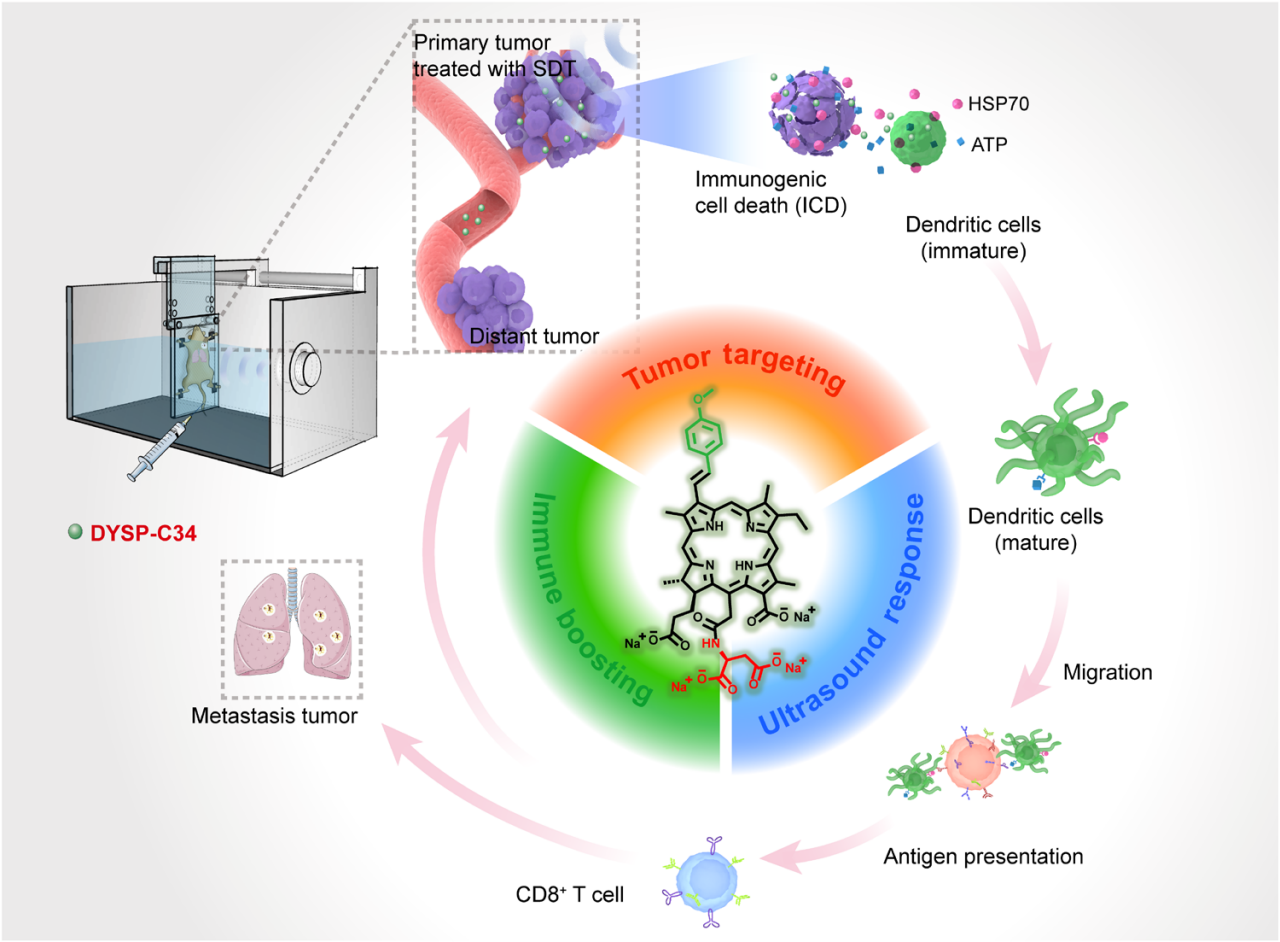
Schematic illustration of the process of the potent US-driven molecular machine, C34, for systemic tumor suppression. C34 molecular machine had preferential tumor accumulation ability, potent US-triggered cytotoxicity, and intrinsic immune-boosting capacity.

Here, we designed a “molecular machine” named DYSP-C34 (abbreviated as C34) by introducing 3^2^-aryl and 15-aspartyl substituents to CHC. Our results revealed that this strategic chemical modification successfully endowed the resultant molecule, C34, with multiple favorable properties, such as improved lipophilic/hydrophilic balance, intensified US-induced ROS production capacity, and better cellular permeability, resulting in the excellent tumor target efficiency and notable SDT-mediated tumor regression. Unexpectedly, C34 itself exhibited mild immunogenicity by stimulating APCs directly. The sono/immunosynergistic effect induced by C34-mediated SDT could profoundly regress tumor systemically (Fig. 1).

## RESULTS

### Preparation and characterization of C34

**Fig. 2.**
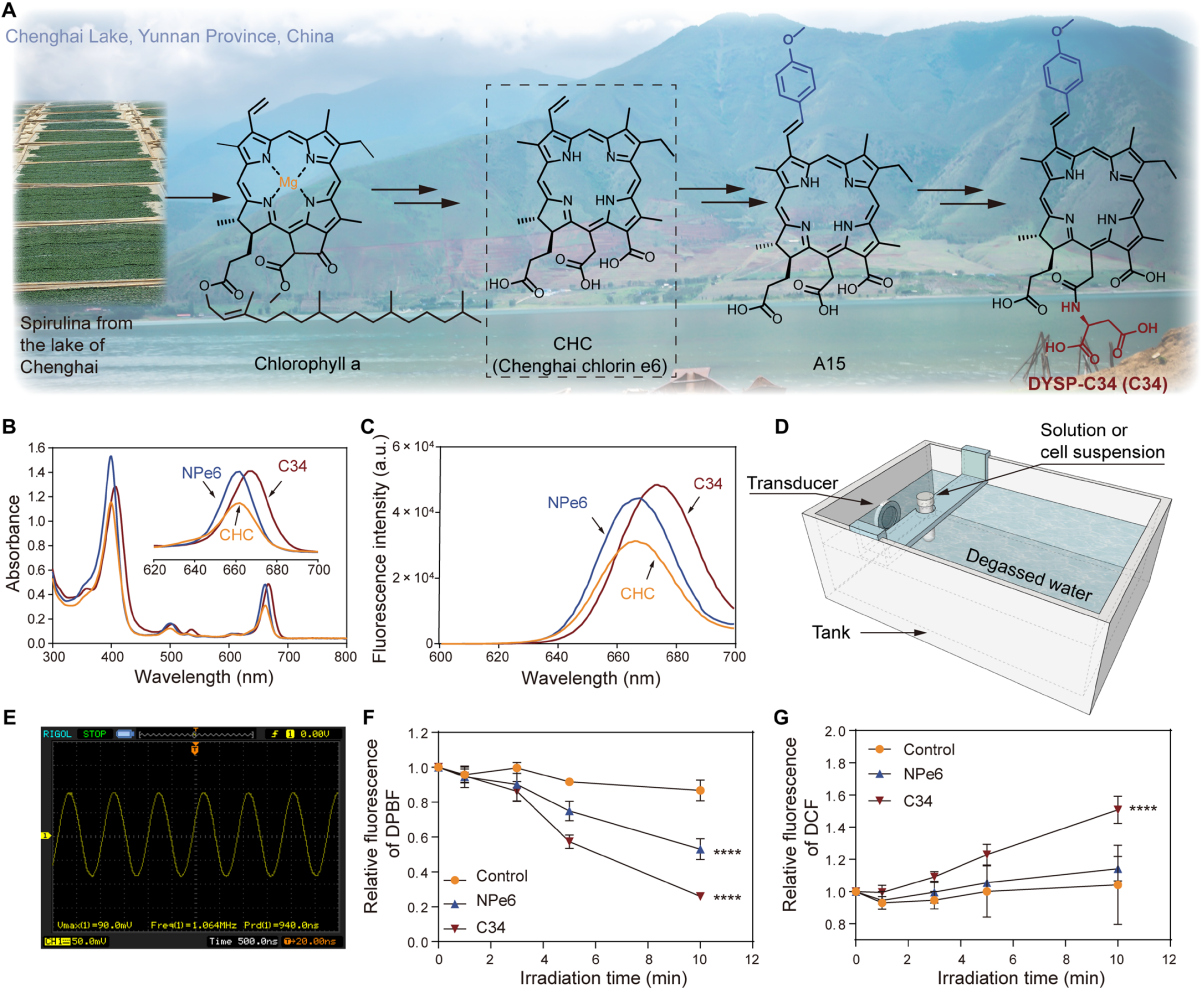
Schematic and characterization of C34. (**A**) Schematic showing the preparation process of C34. (**B** and **C**) UV-vis (B) and fluorescence (C) spectra of CHC, NPe6, and C34. a.u., arbitrary units. (**D**) Illustration of the self-designed experimental configuration that provided the free-field US condition for in vitro study. (**E**) Free-field ultrasonic waveform detected by the hydrophone. (**F** and **G**) Detection of ROS generation capacity of C34 and NPe6 in solution by the probes of DPBF (F) and DCFH (G), respectively, as the US exposure time increased. Data were presented as means ± SD (*n* = 3 per group) and compared using two-way analysis of variance (ANOVA). ****P < 0.0001.

The scheme of preparation of C34 is shown in Fig. 2A. CHC was prepared from chlorophyll a, which was extracted from spirulina powders derived from Chenghai Lake in Yunnan Province, China, as we previously reported (*25*). The synthesis of 3^2^-(4-methoxyphenyl)-15^2^-aspartyl-CHC is described in fig. S1. Briefly, (E)-3^2^-(4-methoxyphenyl)-CHC (A15) was prepared from CHC via Ru-catalyzed olefin metathesis reactions. The aspartic acid side chain was introduced at C-15^2^ regioselectively using 1-ethyl-3-(3-dimethylaminopropyl)carbodiimide hydrochloride (EDCI) as a condensing agent, followed by hydrolysis to yield C34 (fig. S1).

The ultraviolet-visible (UV-vis) absorption spectra of CHC, NPe6, and C34 in methanol are shown in Fig. 2B. The C34 molecule demonstrated a considerable bathochromic shift (7 nm) in both Soret and Qy bands compared with CHC or NPe6. The redshift might be due to the π-extension effect caused by the conjugated connection of the aromatic groups to 3-vinyl of chlorin macrocycle, which had been fully studied in our previous work (*26*). As similarly observed in fluorescence spectra (Fig. 2C), C34 showed distinct emission bands, with a closely 8-nm bathochromic shift in comparison to CHC or NPe6.

To assess the acoustic responsive activity of the C34 molecule, one self-designed low-intensity US irradiation system was set up for in vitro study (Fig. 2D). Currently, the most common US exposure configuration is usually equipped with a transducer at its bottom, in which the medium-containing cell culture dish is placed above the center of the transducer (fig. S2) (*27*–*29*). In this way, US spreads through the bottom of the dish to the water-air boundary on the top (*30*–*32*), resulting in the formation of standing waves (*32*), which are unstable, less reproducible, and much easier for causing cellular mechanical damage (*33*). Thus, to completely eliminate standing waves, we designed an US irradiation configuration with a 1.0-MHz therapeutic transducer (20 mm in diameter) on the side of the tank that was coated with an ultrasonic absorbent material to minimize water-air boundary reflections (Fig. 2D). In addition, under this optimal free-field condition, the pressure amplitude and system temperature could be maintained to be very stable during US exposure (Fig. 2E). The root mean square pressure (*p*_RMS_) amplitude corresponding to different spatial-average temporal-average intensity (*IS*_ATA_) is shown in table S1.

Although the mechanism of ROS generation during SDT remains unclear, ROS is still considered as an important cytotoxic species for the killing effect of SDT (*34*). ROS generation capacity of C34 in the solution was assayed with two commercial ROS-sensing agents, 1,3-diphenylisobenzofuran (DPBF) and 2′,7′-dichlorofluorescin (DCFH). Upon interaction with ROS, DPBF could be bleached to the corresponding diketone, whereas DCFH turned to be its oxidized form, DCF, resulting in the increase of fluorescent signal. As shown in Fig. 2 (F and G), triggered by US, C34 produced significantly higher levels of ROS than NPe6 in solution in a time-dependent pattern. Collectively, the introduction of the π-extension system to the macrocyclic structure of CHC could be a distinct advantage for enhancing acoustic responsive activity, enabling the potential improvement of SDT efficiency for in vivo application.

### In vitro sonocytotoxicity and ICD effect

**Fig. 3.**
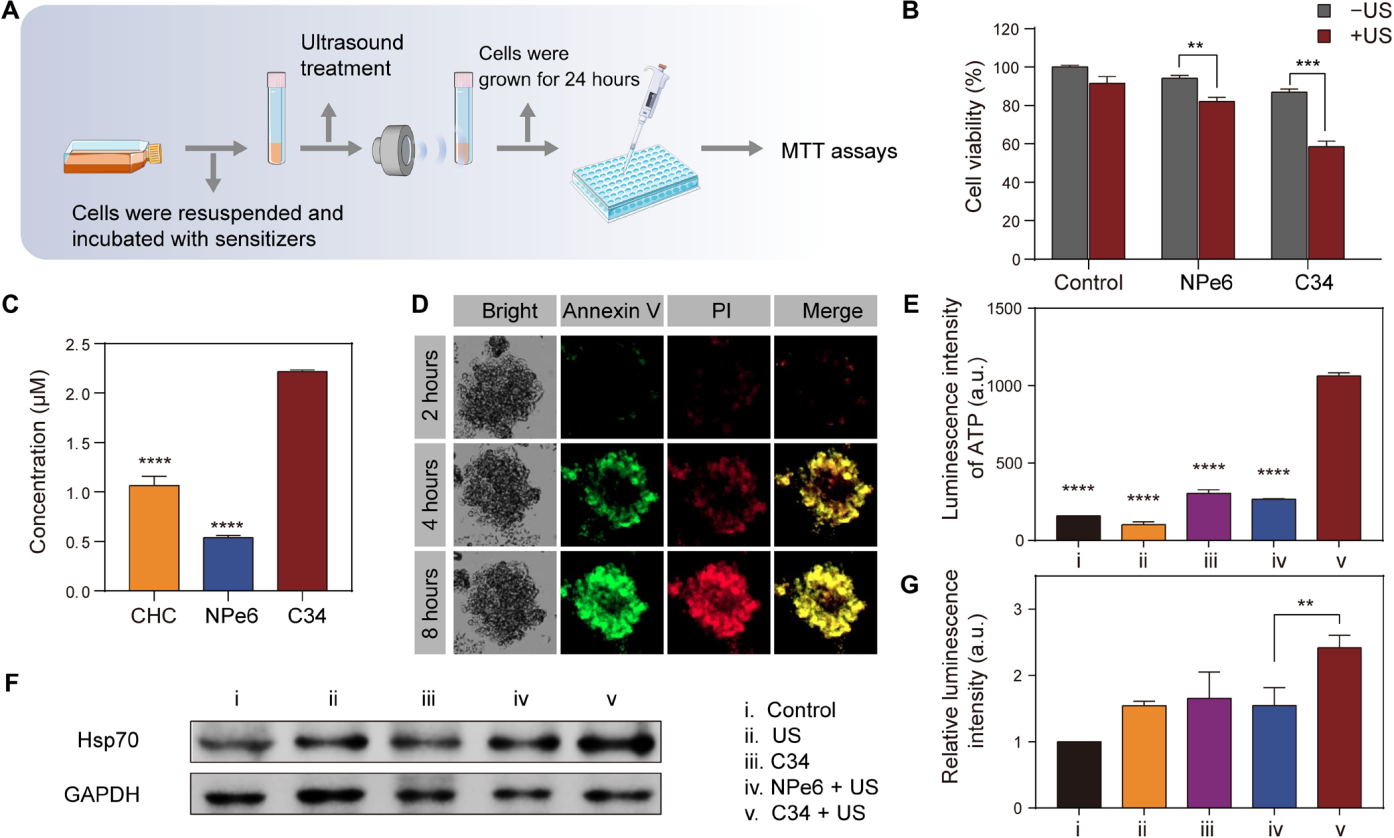
In vitro sonocytotoxicity and ICD effect induced by C34-mediated SDT. (**A**) Illustration of the setup of in vitro sonodynamic experiment. (**B**) Evaluation of sonotoxicity of C34 and NPe6 against MCF-7 cells by MTT assay. (**C**) Cellular uptake levels of CHC, NPe6, and C34 at 25 μM after 0.5 hours of incubation. (**D**) Annexin V/propidium iodide (PI) dual staining of 3D tumor spheroids at 2, 4, and 8 hours after SDT treatment with C34. (**E**) Extracellular ATP levels were detected at 6 hours after various treatments. (**F**) Representative image of Western blot to analyze HSP70 expression in 4T1 tumor cells after various treatments. (**G**) Relative protein levels of HSP70 calculated from Western blot. Data were shown as means ± SD (*n* = 3 per group) and correspond to three independent experiments. Significant statistical analysis was calculated via two-way ANOVA (B) or one-way ANOVA with Tukey’s multiple comparison test (E and G). **P < 0.01, ***P < 0.001, and ****P < 0.0001.

The US-induced cytotoxicity of C34 was evaluated on tumor cells by MTT [3-(4,5-dimethylthiazol-2-yl)-2,5-diphenyl tetrazolium bromide] assay in vitro (Fig. 3A). MCF-7 cells were exposed to the increasing concentrations (0 to 50 μM) of C34 or NPe6 for 0.5 hours and then followed with or without US irradiation. As shown in fig. S3, both treated groups remained up to 85% of the cell viability in the absence of US, indicating that these two molecules had negligible cytotoxicity within the concentration. However, after US irradiation, the survival rates of MCF-7 cells markedly decreased (fig. S3). Note that C34 exhibited remarkably potent US-triggered killing effect than NPe6 (Fig. 3B), which might be associated with the superior ROS generation capacity of C34 as mentioned above. In addition to that, the fine-tuned molecular liposolubility via the introduction of a methoxyphenyl group to the C4 site in the chlorin structure improved the cellular internalization of C34 (Fig. 3C). Treatment of three-dimensional (3D) tumor spheroids with C34 and the analysis of annexin V/propidium iodide (PI) staining after US irradiation showed that the proportion of apoptotic and/or necrotic cells increased from the edge to the core of tumor spheroids as time increased (Fig. 3D), suggesting the good multilayer cellular penetrability of C34. In some extent, SDT drives tumor cells to go through ICD (*35*). As a consequence of those mechanisms, the dying tumor cells release damage-related molecular patterns and tumor antigens, thus triggering immune responses and creating an “antitumor vaccine” (*35*). Adenosine 5′-triphosphate (ATP) molecules released from damaged cells are regarded as a “find-me” signal, causing cytokine production from APCs (*36*, *37*). The high expression of heat shock protein 70 (HSP70) under stress is referred to as a distinct biomarker of ICD as well (*38*). Using a luciferase-based ATP testing probe, we observed abundant ATP secreted from the group treated with C34 + US (Fig. 3E). In addition, HSP70 was remarkably up-regulated in the C34-treated group at 6 hours after US irradiation (Fig. 3, F and G). These observations indicated the higher ICD-inducing efficiency of C34-mediated SDT, leading to the enhanced adjuvanticity of cancer cells.

### Structure-inherent tumor-targeting capacity and pharmacokinetics

**Fig. 4.**
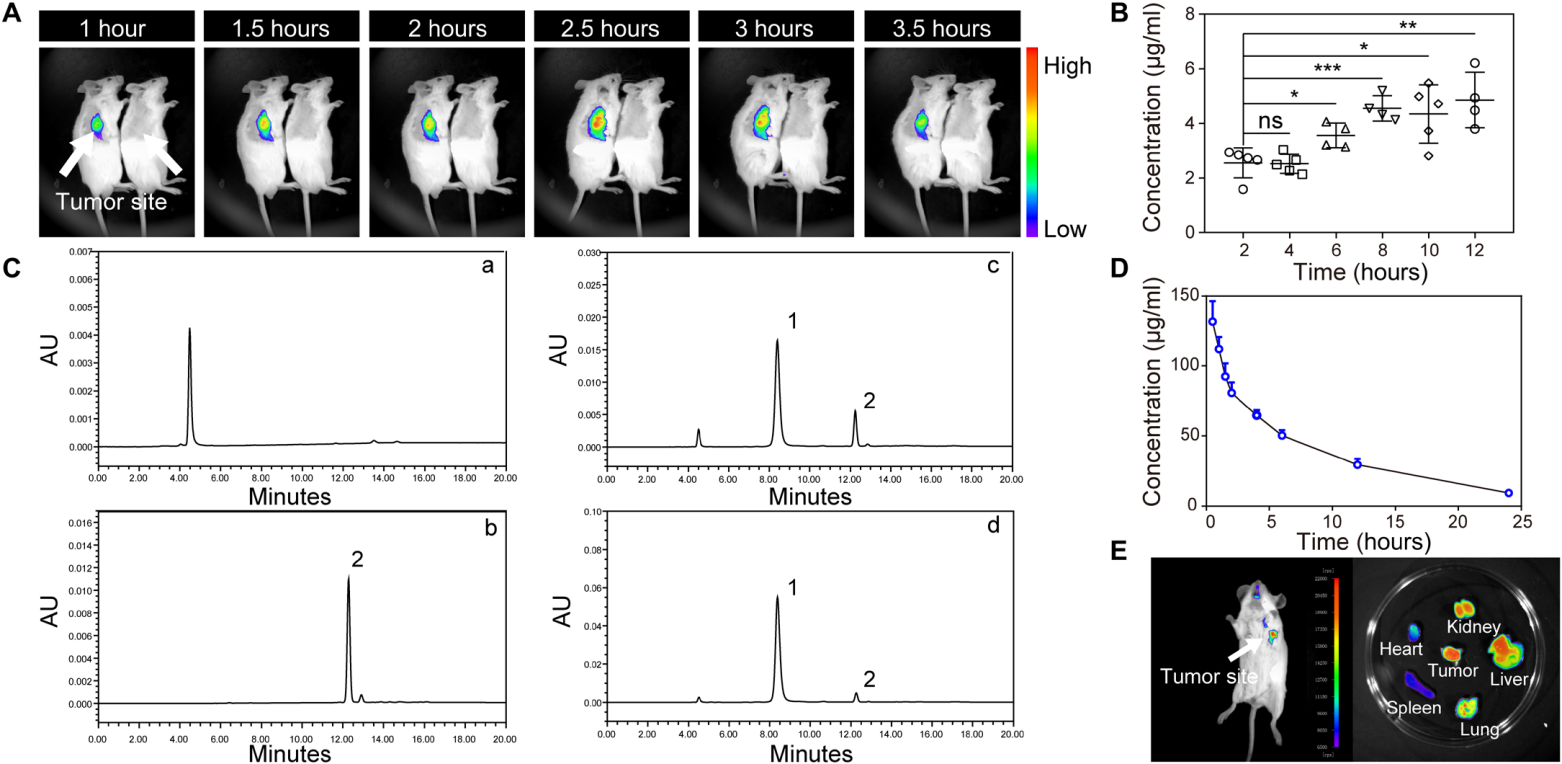
Tissue distribution and pharmacokinetics of C34 in vivo. (**A**) In vivo real-time fluorescence imaging in H22 tumor–bearing mice after intravenous injection of C34 (left) at 16 mg/kg body weight or phosphate-buffered saline (PBS) (right). (**B**) C34 showed increased accumulation in tumor sites as time increased after intravenous injection (16 mg/kg). (**C**) Representative HPLC profiles of (a) blank rat plasma, (b) IS (5 μg/ml), (c) blank rat plasma added with C34 (40 μg/ml) and IS (5 μg/ml), and (d) rat plasma sample collected at 5 min after a single intravenous administration of C34 (16 mg/kg) and added with IS. Peak 1, C34; peak 2, IS. (**D**) Mean plasma concentration-time profile of C34 after single intravenous administrations (16 mg/kg). (**E**) In vivo real-time fluorescence imaging of the orthotopic 4T1 breast cancer model at 2 hours after intravenous injection of C34 (left) and ex vivo imaging of the collected organs (right). Data were shown as means ± SD (*n* = 4 to 5 per group). *P* values were calculated using Student’s *t* test (B). *P < 0.05, **P < 0.01, and ***P < 0.001.

The feature of substantial enrichment into tumor sites is the premise for a drug that could be applied to cancer therapy. The tumor specificity of C34 was first assessed on the xenograft H22 tumor–bearing mouse model. C34 exhibited notable tumor-specific distribution from 1-hour intravenous post-injection assessed by in vivo real-time fluorescence imaging system (Fig. 4A). Consistently, high-performance liquid chromatography (HPLC) quantitative analysis showed increased accumulation of C34 in the tumor tissues as time increased after administration (Fig. 4B), validating the excellent tumor-targeting and tumor-accumulating capacity of C34 in vivo. Similar tendency was observed in the liver but not in other organs (fig. S4 and table S2), suggesting that C34 might undergo the liver metabolic pathway. The prominent tumor-targeting capacity of C34 was further evaluated on the orthotopic 4T1 breast cancer model as well. The ex vivo fluorescence imaging of the collected organs provided the direct evidence of the localization of C34 in the tumor tissue (Fig. 4E). To investigate the pharmacokinetics of C34, plasma samples were collected and analyzed. As shown in Fig. 4C, the peaks of C34 and internal standard (IS) were well separated without any interference. Plasma concentrations of C34 at different time points were determined after intravenous administration at a dose of 16 mg/kg body weight (Fig. 4D). The pharmacokinetic parameters were analyzed by DAS 2.0 software, and the plasma concentration-time profile of C34 was used a noncompartmental mode. Meanwhile, the analyzed data shown in table S3 suggested that C34 could be absorbed by tissues rapidly, resulting in a relatively slow elimination velocity (*t*_1/2z_ = 6.984 hours).

### Systemic tumor suppression

**Fig. 5.**
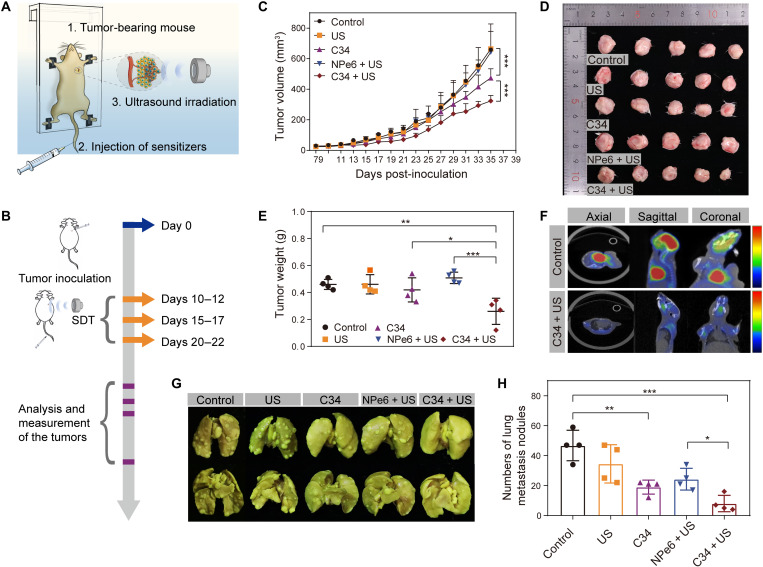
The synergetic inhibitory effect of C34-mediated SDT on primary tumor growth and lung metastasis. (**A**) Illustration of experimental setup for SDT in vivo. (**B**) Schematic illustration of intravenous administration of C34 and US irradiation in the breast cancer–bearing lung metastasis model. (**C**) Tumor growth curves of different groups after various treatments. (**D**) Photographs of excised primary breast tumors at the end of treatments. (**E**) Average weights of excised primary breast tumors. (**F**) Noninvasive monitoring tumor metastasis in the lung by PET/CT (axial, sagittal, and coronal view). (**G**) Representative photographs showing the gross appearance of pulmonary metastatic tumor nodules after fixation with Bouin’s solution (top, frontside; bottom, backside). (**H**) Average numbers of tumor nodules in the lungs of different treated groups. Data correspond to four independent experiments and were shown as means ± SD [*n* = 5 per group (C and D); *n* = 4 per group (E and H)]. *P* values were calculated using two-way ANOVA (A), one-way ANOVA with Dunnett’s multiple comparison test (E), or Student’s *t* test (H). *P < 0.05, **P < 0.01, and ***P < 0.001. Photo credit (D and G): Liu Wang, Department of Pharmacy, Dalian University of Technology.

The in vivo antitumor effect of C34-mediated SDT was first assessed in the model of breast cancer with lung metastasis. The mouse 4T1 breast tumor is poorly immunogenic, highly tumorigenic, and invasive (*39*). Analogous to human mammary cancer, the 4T1 tumor can spontaneously metastasize from the primary tumor in the mammary gland to multiple distant sites including lymph nodes, blood, lung, and bone (*40*), which makes it a suitable experimental animal model for evaluating the therapeutic effect against both primary and metastatic tumors simultaneously. For in vivo treatment of SDT, a low-intensity nonfocused US irradiation system was set up as illustrated in Fig. 5A. Usually, the mouse, except its head, was taped on the mouse holder and immersed in a degassed water bath along with the US transducer, which faced the tumor inoculation site of the mouse.

Here, the breast cancer model with lung metastasis was established by subcutaneous injection of 3 × 10^5^ 4T1 cells into the second pair of mammary glands of female BALB/c mice (day 0). Once the primary tumor volume reached ~50 mm^3^, tumor-bearing mice were randomly divided into the following five groups: control, US, C34, NPe6 + US, and C34 + US. For SDT-treated groups, the mice were intravenously injected with C34 or NPe6 (16 mg/kg body weight) and followed by US irradiation (1.064 MHz, 1.88 W/cm^2^, 30 min) at 4 hours after administration of sonosensitizers. Each group received three consecutive treatments from days 10, 15, and 20, respectively (Fig. 5B). As shown in Fig. 5 (C to E), neither US alone nor NPe6 + US induced an antitumor effect on solid tumor growth, indicating that NPe6 + US treatment completely failed to control primary tumor development. By contrast, we observed a pronounced inhibition in tumor progression in C34 + US–treated group, which, on day 35, had a mean tumor volume of 320 mm^3^ compared with 660, 664, 470, and 643 mm^3^ for the animals treated with phosphate-buffered saline (PBS), US, C34, and NPe6 + US, respectively. Unexpectedly, the primary solid tumor inhibitory efficacy was found to be markedly improved by treating with C34 alone, even superior to NPe6-mediated SDT (*P* = 0.004; Fig. 5C). Similar but more pronounced effects were also observed when lung metastases were analyzed. At the end of the experiment, mice were euthanized and assessed for the extent of metastasis to lungs by examining the gross appearance of pulmonary nodules after fixation with Bouin’s solution for 24 hours. Lungs of mice in the control group displayed multiple apparent tumor nodules with various sizes on the surface (Fig. 5G, top, frontside; bottom, backside), whereas the C34 + US–treated group showed very few visible tumor nodules in the lung, indicating the substantially slower progression of metastasis compared with the other groups (Fig. 5, G and H, and fig. S5). We further conducted in vivo imaging by using positron emission tomography–computed tomography (PET/CT) for small animals, which is typically considered as an excellent approach for detecting micrometastatic lesions due to its higher anatomical resolution (*41*, *42*). As shown in axial, sagittal, and coronal PET/CT images (Fig. 5F), intensive uptake of ^18^F-fluorodeoxyglucose (^18^F-FDG) was observed in the lung of control mice, while negligible ^18^F-FDG signal could be seen in that of the C34 + US group, confirming the significant reduction of metastatic disease by C34-mediated SDT. Collectively, all of these results indicated the excellent performance of C34-mediated SDT in synergistic control of primary tumors and a significant reduction in spontaneous lung metastases. During the whole therapeutic course, all mice did not have noticeable abnormal body weight changes (fig. S6), and no obvious destructive cell necrosis or inflammation lesions in major organs including the heart, liver, spleen, and kidney were observed in histological analysis (fig. S7), thereby implying the high biocompatibility and safety of C34-mediated SDT for biomedical applications.

### Potentiation of the adaptive antitumor immune responses

**Fig. 6.**
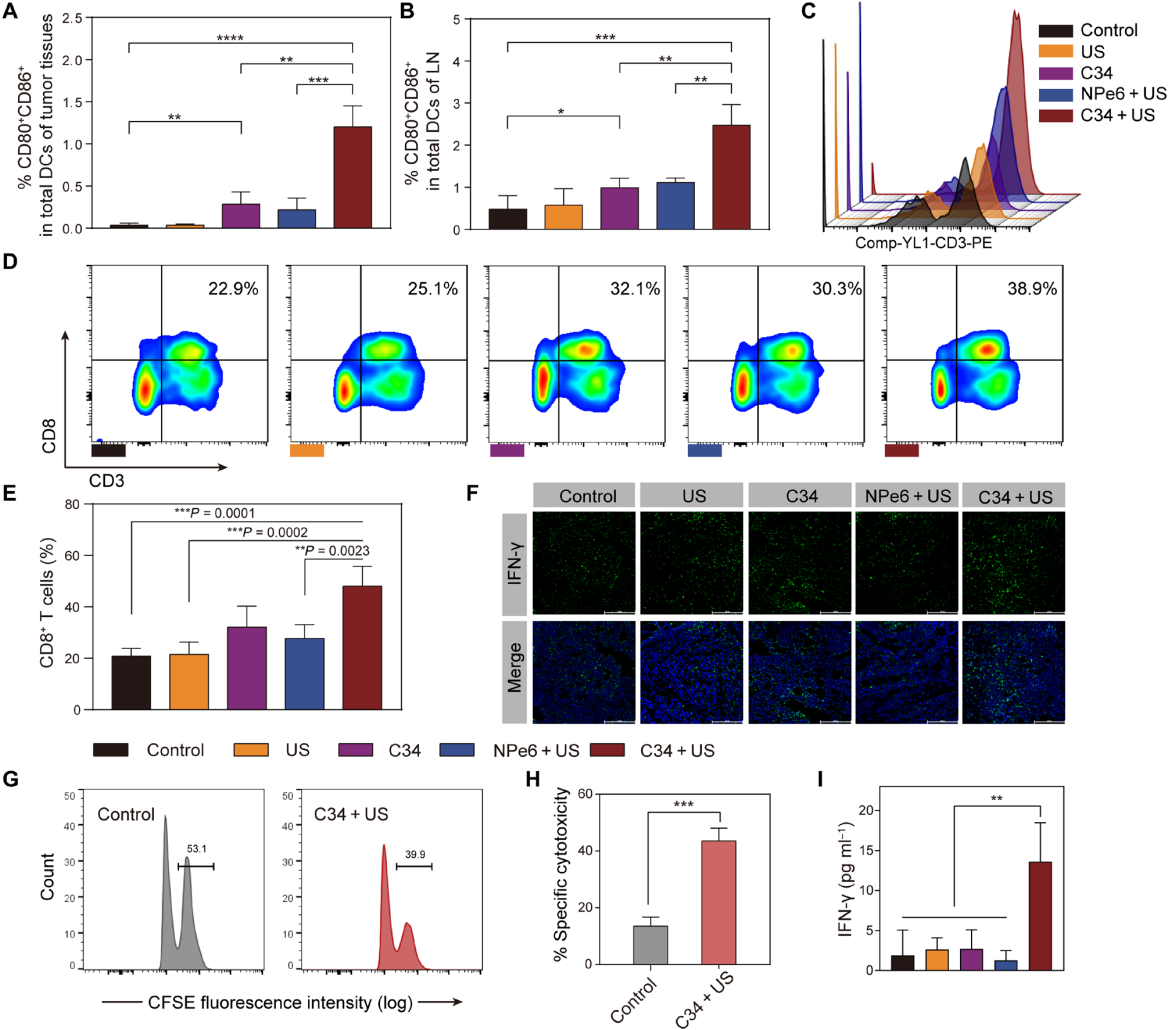
C34-mediated SDT potentiated the adaptive antitumor immune responses in the 4T1 breast cancer model with lung metastasis. (**A** and **B**) Percentages of mature DCs (CD11c^+^CD80^+^CD86^+^) in tumor in situ (A) and tumor-draining lymph nodes (B) were analyzed by flow cytometry after different treatments. (**C**) Representative histogram profiles of CD3^+^ cells in the tumor tissues of different treated groups. (**D**) Representative profiles of flow cytometry showing the percentages of infiltrated CD8^+^ T cells in the tumor tissues of different groups. (**E**) Proportions of the infiltrated CD8^+^ T cells in the primary tumors. (**F**) Immunofluorescence analysis of IFN-γ production on the tissue sections of the collected primary tumors. Scale bars, 200 μm. (**G** and **H**) Proportions of CFSE^high^-OVAI–pulsed target cells lysed by effector CD8^+^ CTLs in the splenocytes of C34 + US–treated or control group were determined by in vivo cytotoxicity assay. (**I**) IFN-γ levels in the serum of different treated groups, determined by enzyme-linked immunosorbent assay (ELISA). Data were shown as means ± SD [*n* = 3 per group (A, B, E, and I); *n* = 4 per group (G and H)], corresponding to four independent experiments, and were compared with Student’s *t* test (A, B, and H) or one-way ANOVA with Tukey’s multiple comparison test (E and I). *P < 0.05, **P < 0.01, ***P < 0.001, and ****P < 0.0001.

To understand the mechanisms underlying the superior systemic antitumor therapeutic efficacy, we analyzed the maturation status of dendritic cells (DCs) and the infiltration of cytotoxic CD8^+^ T lymphocytes (CTLs) primed by C34-mediated SDT in the breast cancer model with lung metastasis (*43*). Three days after the final treatment, the primary solid tumor and apparently enlarged ipsilateral tumor axillary draining lymph nodes (DLNs) were excised and stained for flow cytometry analysis. C34-mediated SDT resulted in significant increased levels of DC activation and maturation (CD11c^+^CD80^+^CD86^+^) in the tumor tissue and DLNs (Fig. 6, A and B, and fig. S8). Specifically, 5.5- and 2.2-fold more activated DCs were present in tumor tissues and DLNs, respectively, by C34-mediated SDT, compared with the NPe6 + US group. As a consequence, C34 + US led to substantial expansion and infiltration of CD8^+^ T cells in the tumor tissue, indicating the generation of a more robust CD8^+^ T cell response (Fig. 6, C to E). The antitumor functions of these CD8^+^ T cells were further confirmed by intracellular interferon-γ (IFN-γ) staining and in vivo cytotoxicity assay. IFN-γ has been widely recognized for its proinflammatory capability in mediating antitumor immunity (*44*). Consistently, a higher amount of IFN-γ production was found in the tumor tissue sections from C34 + US–treated group (Fig. 6F), which, by large, originated from the tumor-infiltrating CD8^+^ T cells because they were reported as the main producer of IFN-γ (*45*). The in vivo cytotoxicity assay showed the significant lysis (up to 42%) of CFSE^high^–ovalbumin I (OVAI)–pulsed target cells, suggesting the antigen-specific cytolytic effect of those effector CD8^+^ CTLs primed by C34 + US treatment (Fig. 6, G and H). In addition, the serum level of IFN-γ was remarkably elevated in the C34 + US–treated group compared to other treated or control group (Fig. 6I). Together, these data supported the idea that, in addition to the direct tumoricidal effects, the outperformance of C34-mediated SDT in controlling both primary and metastatic tumors might be attributed to a successful augment of DC recruitment, maturation, and cross-presentation, leading to strong potentiation of tumor antigen–specific T cell responses.

### Intrinsic immune-boosting capacity of C34

**Fig. 7.**
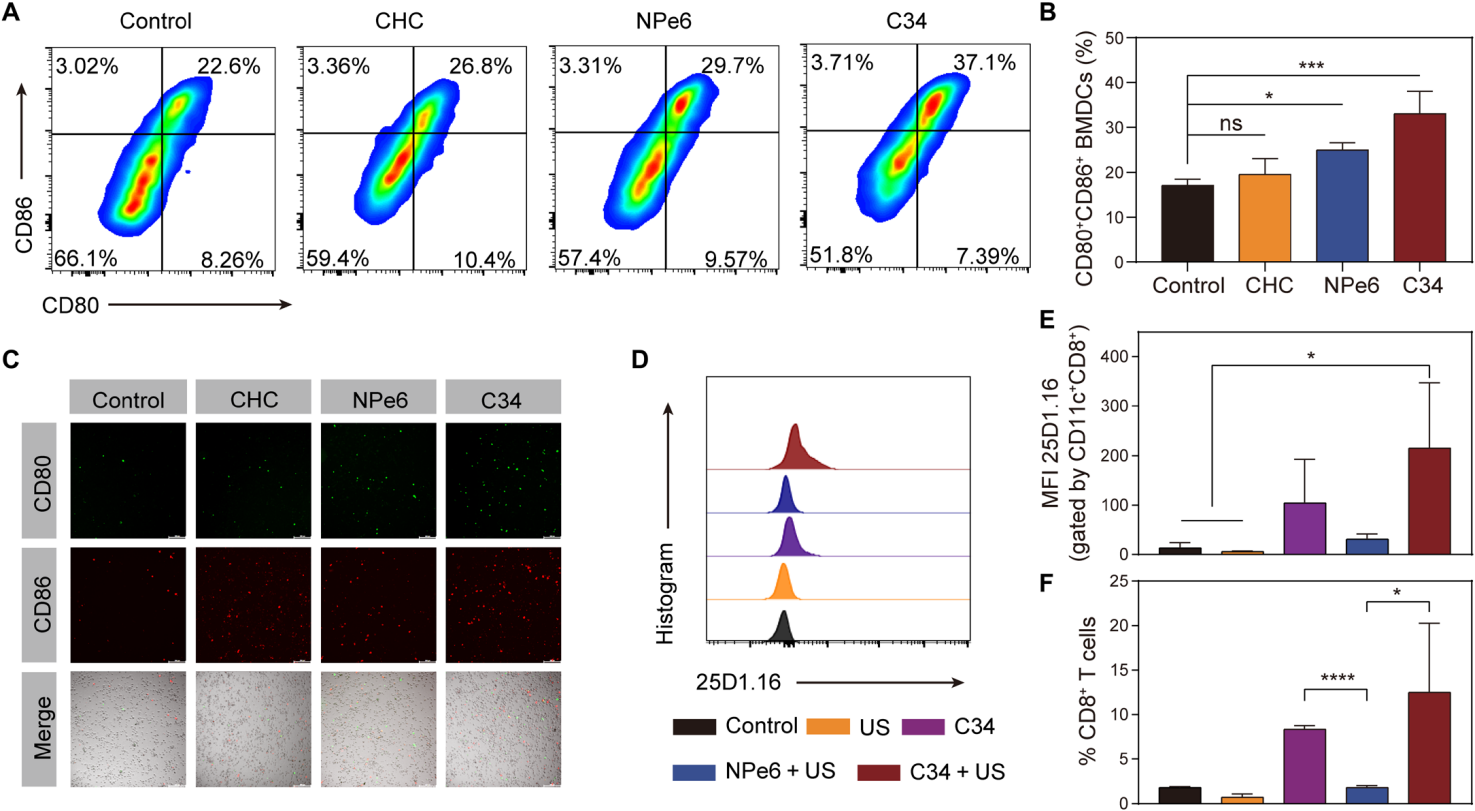
C34-mediated SDT elicited antigen-specific CD8^+^ CTL response. (**A** and **B**) Representative profiles (A) and proportions of matured DCs (CD11c^+^CD80^+^CD86^+^) were analyzed by flow cytometry after being cocultured with different sonosensitizers for 6 hours (B). (**C**) Fluorescence imaging of matured DCs stained with CD80-FITC and CD86-allophycocyanin after incubation with different sonosensitizers. Scale bars, 200 μm. (**D** and **E**) Mean fluorescence intensity (MFI) of 25D1.16 antibody labeling on CD8^+^ BMDCs (gated on CD11c^+^CD8^+^ DCs) in the cellular cocultured system. (**F**) Proportions of OVAI antigen–specific CD8^+^ T cells cross-primed by 25D1.16^+^CD8^+^ DCs in the cellular cocultured system. Data were shown as mean % ± SD [*n* = 4 per group (B, E, and F)] and were compared with one-way ANOVA with Tukey’s multiple comparison test (B and E) or Student’s *t* test (F). *P < 0.05, ***P < 0.01, and ****P < 0.001. ns, not significant.

DCs are the main professional APCs for induction of T cell–adaptive responses (*46*). C34 treatment alone exhibited notable DC maturation and relative higher tumor infiltration of CD8^+^ T cells in comparison with the control group (Fig. 6, A to E), which might be the reason to explain why C34 was able to control the tumor progression to some extent in the absence of US (Fig. 5, C, E, and H). Given the impossibility of C34 inducing direct cell killing effect without US, we asked whether there was some intrinsic property governed by C34 having an impact on DCs. To elucidate this hypothesis, immature bone marrow–derived DCs (iBMDCs) were used to evaluate the stimulation effect by analyzing the up-regulations of costimulatory molecules CD80/CD86. Unexpectedly, when the iBMDCs were cocultured with NPe6 or C34 for 6 hours, in the absence of US irradiation, the proportion of mature DCs (CD80^+^CD86^+^) induced by C34 was 1.3-fold higher than that of the NPe6-treated group (Fig. 7, A and B). The quantitative data confirmed that C34 could significantly facilitate the maturation of DCs in vitro, whereas CHC showed no obvious immune stimulation effect (Fig. 7B). Consistent with these observations, the results of fluorescence imaging showed higher CD80 (green) and CD86 (red) signals in the C34-treated group (Fig. 7C).

We next investigated the tumor antigen cross-priming by DCs in vitro because it is a key process for inducing the generation of CTLs with specificity for tumor antigens (*47*). B16-OVA cells were co-incubated with iBMDCs and OT-1 naive CD8^+^ T cells after being exposed to US, C34, NPe6 + US, or C34 + US. Notably, C34 + US treatment induced a significantly up-regulated expression of OVAI-peptide-MHC (major histocompatibility complex) class I complexes (stained with 25D1.16 antibody) on CD8^+^ BMDCs (Fig. 7, D and E), consequently resulting in the activation and proliferation of OVAI antigen–specific CD8^+^ T cells (Fig. 7F). In consistency with the aforementioned observations, C34 itself could also initiate this cascade, although the activation levels were lower than in the C34 + US–treated group, which might be attributed to less tumor antigen exposure in the absence of US. Collectively, these results confirmed the intrinsic immune-boosting capacity of C34 and its potential synergistic role during SDT.

### Antimetastasis efficiency in aggressive liver metastatic model

**Fig. 8.**
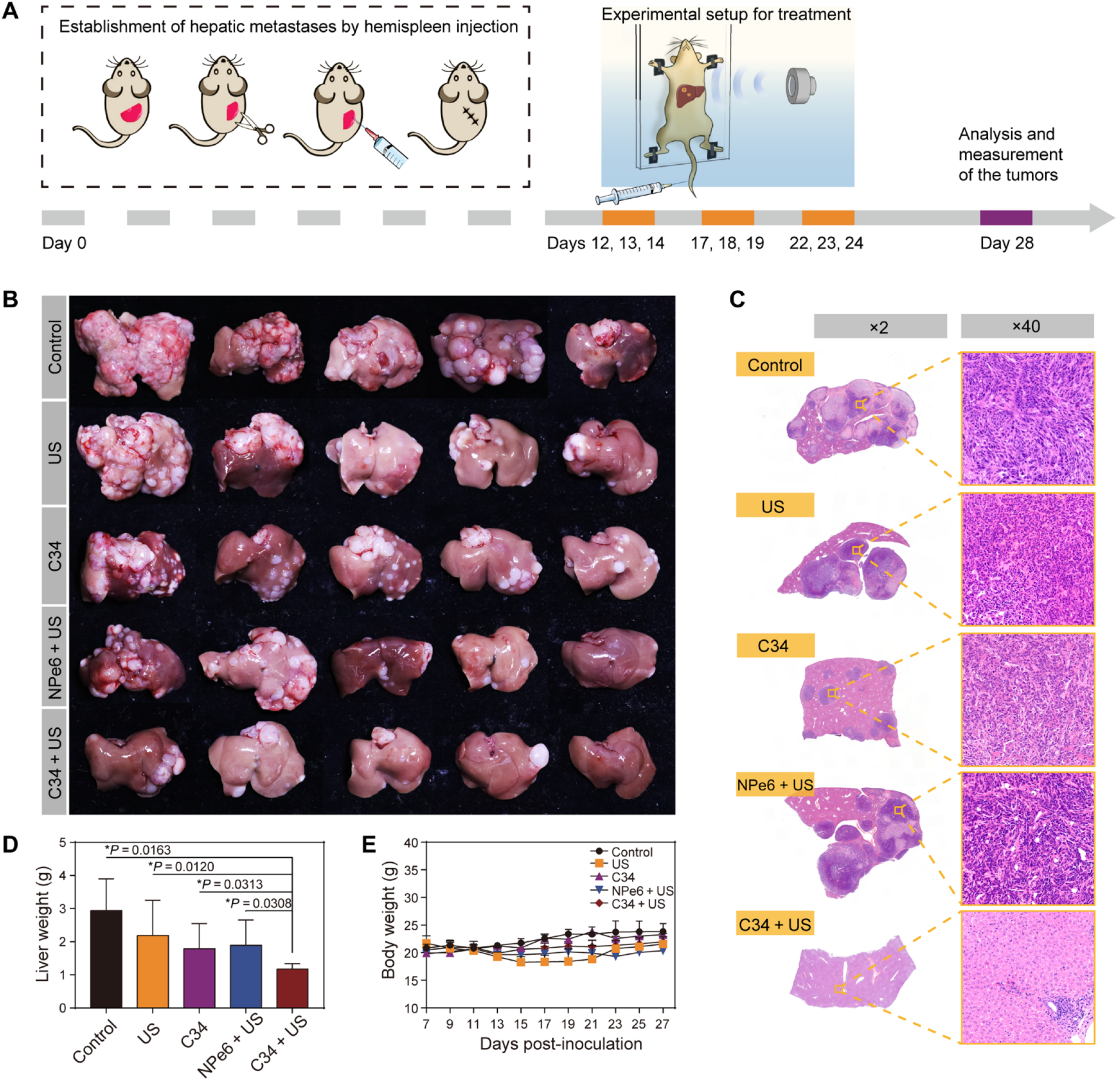
Antitumor effect of C34-mediated SDT evaluated in the colorectal cancer model with liver metastases. (**A**) Schematic illustration of the establishment of murine hepatic metastases by hemispleen injection and the experimental setup for SDT in vivo. (**B**) Photographs of liver tissues from different treated groups. (**C**) Hematoxylin and eosin staining of the liver sections (magnifications: ×2 and ×40). (**D**) Average weights of the livers at the end of treatments. (**E**) Body weight curve versus the number of days after different treatments. Data were presented as means ± SD (*n* = 5 per group) and correspond to two independent experiments. *P* values were calculated via one-way ANOVA with Dunnett’s multiple comparison test. *P < 0.05. Photo credit (B): Liu Wang, Department of Pharmacy, Dalian University of Technology.

The synergistic cytotoxic and immune-boosting effects induced by C34-mediated SDT were further corroborated in a murine colorectal cancer model with hepatic metastases using hemisplenectomy. As indicated in Fig. 8A, the mouse spleen was divided into two separate hemispleens; afterward, one of which was injected with CT26 colon cancer cells and then excised after allowing the seeding of the tumor cells within the liver. Unlike a splenectomized model whose immune system got compromised, in the hemispleen model, the remaining non–tumor-bearing hemispleen could maintain the animal in an immunocompetent condition and the splenocytes could be harvested for further analysis. The advantage of this tumor model was, in a more clinically relevant context, mimicking colorectal cancer metastasis and recurrence in the liver after surgical resection of a primary colonic tumor (*48*). During SDT, the mouse was taped on the mouse holder and immersed in a degassed water bath (Fig. 8A), with an US transducer facing the liver site of the mouse. On day 28 after tumor inoculation, qualitative images of excised livers showed the gross tumor metastases in the control group, indicating the highly aggressive progression of liver metastases in this model (Fig. 8B). Treatment with C34 alone or with NPe6 + US was insufficient to delay the formation of metastatic lesions in livers. However, C34 + US was found to have a much greater efficacy compared with the other treatments (Fig. 8B). Furthermore, mice treated with C34 + US exhibited a considerably lower liver weight in comparison to that of the other treated groups (Fig. 8D). The livers were then sectioned and stained with hematoxylin and eosin for the analysis of microscopic hepatic tumor burden (Fig. 8C). Massive tumor foci were observed in control-, US alone–, and NPe6 + US–treated groups, whereas the tiny micrometastatic tumor lesions were visible in the C34 + US–treated group. The percentage of tumor area occupied in the healthy liver tissue of the C34 + US–treated group was only 2.2%, much lower compared to PBS (78.6%)–, US (72.7%)–, C34 (30.1%)–, and NPe6 + US (72.1%)–treated groups. These data confirmed the synergistic effect of C34-mediated SDT for controlling the aggressive tumor metastasis. During the therapeutic course, a slight decrease in body weight was observed when US was applied, but it was back to normal once US treatment was finished, implying the negligible toxicity caused by the treatments (Fig. 8E).

### Systemic antitumor immunity induced by C34-mediated SDT

**Fig. 9.**
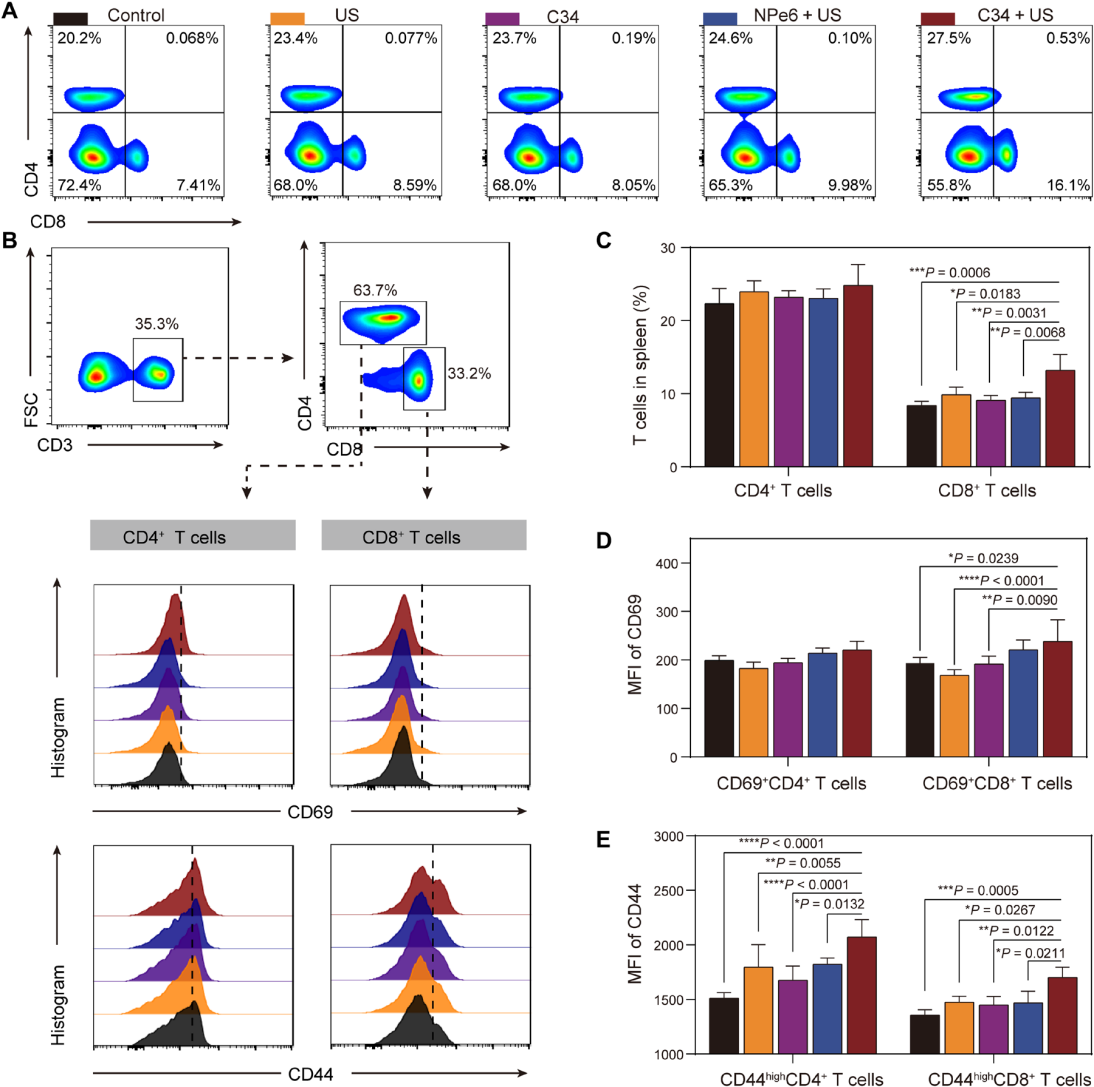
Systemic antitumor immunity induced by C34-mediated SDT in the colorectal cancer model with liver metastases. (**A**) Representative flow cytometry plots showing different groups of CD8^+^ and CD4^+^ T cells in spleen. (**B**) Flow cytometry plots showing percentages of CD69^+^ or CD44^+^ cells (gated on CD4^+^CD3^+^ or CD8^+^CD3^+^ cells) in the spleen after various treatments. FSC, forward scatter. (**C**) Proportions of CD4^+^ T cells and CD8^+^ T cells in the spleen (gated on CD3^+^ cells). MFI of CD69 (**D**) and CD44 (**E**) marker expression on CD4^+^ and CD8^+^ T cells in the splenocytes (gated on CD3^+^ cells). Data were shown as mean % ± SD [*n* = 4 per group (C to E)] and correspond to two independent experiments. Significant statistical analysis was calculated via two-way ANOVA. *P < 0.05, **P < 0.01, ***P < 0.001, and ****P < 0.0001.

To test whether the immune effect induced by C34-mediated SDT was systemic, T cell profiling in the spleen was analyzed in the colon cancer with the liver metastatic model after various treatments. As shown in Fig. 9 (A and C), compared with the control or NPe6 + US group, C34 + US led to a 2.17- or 1.61-fold increase in CD8^+^ T cells, suggesting the expansion and proliferation of CTLs in periphery, which are considered as the major contributor to drive direct cytotoxic killing of tumor cells (*49*). The percentage of CD4^+^ T cells was also slightly increased by C34 + US treatment, but not significantly different from the other treated groups (Fig. 9C), which could be a result of the more predominant CD8^+^ T cell response induced by C34-mediated SDT. CD69 is known to be a classical early marker of lymphocyte activation (*50*, *51*). We next evaluated the expression of CD69 to assess the activated status of T cells from the treated mice. Consistently, C34 + US was found to induce a considerable increase in the percentage of CD69^+^CD8^+^ T cells, but not significant in CD69^+^CD4^+^ T cells, in comparison to the other treated groups (Fig. 9, B and D). In addition, CD8^+^ T cells primed by C34-mediated SDT exhibited a marked increase of an effector/memory CD8^+^ subset expressing high levels of CD44 cell adhesion molecule (Fig. 9, B and E). Consistently, C34 + US induced higher differentiation of CD4^+^ T cells into the memory phenotypes (CD44^hi^CD4^+^) (Fig. 9, B and E), indicating the potential of generating a long-lasting immune memory to prevent tumor relapse (*52*).

## DISCUSSION

Emerging evidences have indicated that systemic tumor suppression requires combinational therapeutic strategies, which usually lead to better clinical outcomes than monotherapies for cancer treatment (*53*). However, combinational therapies may increase the complexities of treatment and stress patients by bringing in multiple agents and regimens (*54*). Therefore, in this work, to address the pressing clinical need for developing a simple yet effective therapeutic approach in regressing primary tumor and blocking metastasis simultaneously, we explored a potent, biocompatible, and multifunctional molecular machine, C34, with preferential tumor accumulation capacity, US-activable cytotoxicity, and intrinsic immune-boosting effect.

The unique chemical structure might be the key feature favoring the tumor accumulation of C34. There have been many studies suggesting the tumor specificity of the chlorin-based sensitizers (*55*), largely due to their high plasma protein binding ability (*56*). C34, as a derivative of CHC and an analog of hematoporphyrin, exhibited a good affinity with the highly metabolic tumor tissue by being carried by the plasma proteins to the tumor vascular system. Moreover, amphiphilicity has been reported as an important factor that affects the tumor accumulation of porphyrin dyes as well (*57*, *58*). The excellent permeability of the C34 molecule within the tumor cells was attributed to the increased liposolubility by introducing the aromatic group to the CHC molecule. The amino acid residue of C34 served as a hydrophilic group to obtain the hydrophilic-lipophilic balance. In addition, cancer cells have a higher magnitude of membrane potential than normal cells (*59*). C34 with positive charges at the carboxyl groups was preferentially prone to accumulate and stay within the tumors.

The intrinsic immune-boosting activity could be another one of the most attractive features of the C34 molecule. Some literature has reported that chlorophyll a, a precursor molecule of C34, could modulate immunity through DCs; however, it lacks direct evidence (*60*). Our observations confirmed that C34 itself was able to trigger antitumor immunity by directly stimulating DCs, leading to the generation of CD8^+^ CTL responses. In addition, this effect could be significantly amplified once being exposed under US irradiation during SDT, which might be due to more extensive tumor antigen exposure. Notably, 3^2^-aryl substituent was the only difference in the chemical structures between Npe6 and C34, resulting in distinct diversity in the property of molecular immunogenicity. This phenomenon prompted us to ask whether the mild immunogenicity of the C34 molecule was associated with the introduction of the π-extension system to the macrocyclic structure of CHC, as we discussed previously (*25*, *26*). In addition, we have performed the reverse docking studies to look for the potential target proteins of C34 related to the DC stimulation. Further biological assays are needed to confirm the mechanism of action of the C34 molecule. Studies on the structure-activity relationship associated with the immune-boosting effect will be undertaken as well, which may provide new insights into the development of more potent sonosensitizers for cancer therapy.

In summary, these satisfactory features of C34 allow a unimolecule-mediated sono/immunosynergistic therapy to become more realistic. C34-mediated SDT could be universally applied to different cancer types, offering a more facile treatment option to clinic.

## MATERIALS AND METHODS

### Experimental design

In this study, a potent, biocompatible, and US-triggered multifunctional molecular machine, named C34, was designed and synthesized. The acoustic responsive activity of C34 was evaluated in vitro. The antitumor efficacy of C34-mediated SDT was assessed in the models of breast cancer with lung metastasis and colon cancer with liver metastasis. Immunological analysis was performed to test the intrinsic immune-boosting capacity of C34.

### Materials

DPBF and DCFH diacetate (DCFH-DA) were purchased from Sigma-Aldrich. Dimethyl sulfoxide and ethanol were purchased from Sinopharm Chemical Reagent Co. Antibodies against cell surface markers for flow cytometry [fluorescence-activated cell sorting (FACS)] assay were purchased from BioLegend.

### Acoustic pressure amplitude measurements

The total acoustic power generated by the given source was measured with an ultrasound power meter (UPM) (UPM-DT100N, Ohmic Instruments, USA). The acoustic wave intensity *I*_SATA_ was calculated according to the formula

$$I_{\text{SATA}}=\frac{P_{A}}{S}$$ (1)

where *P*_A_ is the total acoustic power measured by the UPM and *S* is the active aperture of transducer.

A polyvinylidene difluoride (PVDF)–type hydrophone (HNR-0500; calibration range, 250 kHz to 10 MHz) was placed vertically at a distance of 10 mm from the sensor surface to record the ultrasonic signals. The hydrophone was connected to a digital oscilloscope for the observation, measurement, and processing of the spectrum. To take into account pressure amplitude variations during the ultrasonic duration, the root mean square pressure value *p*_RMS_ was calculated as (*61*)

$$p_{\text{RMS}}=\frac{V_{\text{RMS}}}{M}$$ (2)

where *V*_RMS_ represents the root mean square voltage value at the hydrophone and *M* is the hydrophone sensitivity (V/Pa).

### Determination of ROS production

A mixed solvent [ethanol/water, 1:1 (v/v)] containing C34 or NPe6 (10 μM) and DPBF (20 μM) was prepared. The solutions were then irradiated by US for 10 min. Aliquots were taken at specific time points, and fluorescence at 460 nm (excitation = 417 nm) was recorded with a fluorescence microplate reader. Control group in the absence of any sonosensitizer (i.e., DPBF + US) was performed for comparative purposes.

For the test of H_2_O_2_, the activated DCFH was prepared from DCFH-DA (*62*). The sonosensitizer was then added to the neutralized DCFH (20 μM) solution in an equal volume of ethanol with a final concentration of 10 μM. The mixture solution was prepared just before the beginning of US irradiation, which aimed to minimize the DCFH self-oxidation process. Under free-field US condition, DCFH immediately reacted with formed H_2_O_2_, oxidized to DCF, a highly fluorescent species with an emission peak at 525 nm (excitation = 488 nm). Control group without adding any sonosensitizer was also performed for comparative purposes.

### Cell culture and animals

MCF-7 human breast cancer cells and 4T1 murine breast cancer cells were obtained from the National Infrastructure of Cell Line Resource (Beijing, China). CT26 murine colon cancer cells were purchased from KeyGEN BioTECH Corp. Ltd. (Nanjing, China). These cells were incubated on the cell culture plate in Dulbecco’s modified Eagle’s medium (DMEM) (HyClone) supplemented with 10% fetal bovine serum (PAN-Seratech) and 1% penicillin-streptomycin (HyClone) at 37°C in a humidified, 5% CO_2_ atmosphere. Male C57BL/6 mice (6 to 8 weeks, 18 to 20 g), female BALB/c mice (6 to 8 weeks, 18 to 20 g), and male ICR mice (6 to 8 weeks, 25 to 30 g) were purchased from Liaoning Changsheng Biotechnology (Benxi, China). B16-OVA (OVA-transfected B16F10) cells and the splenocytes of OT-1 mice containing transgenic insert for mouse Tcra-V2 and Tcrb-V5 genes were gifts from H. Gao of Sichuan University, China. All animal experimental protocols in this study were performed after being approved by the Animal Research Ethics Committee of Dalian University of Technology.

### Cellar uptake

MCF-7 cells were resuspended at densities of 5.4 × 10^5^ cells/ml in a 2-ml culture tube. The cells were then exposed to a 25 μM solution of different sonosensitizers in serum-free medium for 0.5 hours in the dark. After incubation, the cells were washed two times with 1 ml of PBS and finally lysed with 1 ml of methanol to release the intracellular sonosensitizers. Quantitative concentration was calculated from the standard curve of concentration-dependent fluorescence intensity.

### In vitro sonocytotoxicity assay

MCF-7 cells in the exponential phase were resuspended with serum-free medium at cell densities of 5.4 × 10^5^ cells/ml. Then, all samples were randomly divided into four groups: control, C34 alone (C34), US alone (US), and C34 + US. For C34 and C34 + US groups, the cells were incubated in the medium containing C34 solution in the dark. After 0.5 hours, the cells were harvested and washed twice by PBS. MCF-7 cells were resuspended at a concentration of 3 × 10^5^ cells/ml in DMEM (serum free) and transferred into a cylindrical polystyrene tissue culture tube for US irradiation. US and C34 + US groups were then treated with free-field US (1.064 MHz, 3.21 W/cm^2^) for 10 min. After various treatments, the cells were placed into 96-well plates and grew for 24 hours. Cell viability was estimated by the standard MTT assay.

Compared to 2D cell cultures, the 3D tumor spheroids can provide a more accurate tumor-mimicking microenvironment. First, agarose [1.5% (w/v)] was dissolved in DMEM and casted in 96-well plates to form molds with homogeneous circular recesses. Next, MCF-7 cell suspension (100 μl; 3 × 10^4^ cells/ml), corresponding to 3000 total cells per well, was incubated at 37°C for 24 hours. Afterward, DMEM containing 50 μM C34 was added to each well and incubated in the dark at 37°C for another 24 hours. Following incubation, the spheroids were transferred to a cylindrical polystyrene tissue culture tube for exposure to US. After exposure, the spheroids were washed with PBS before placing into 96-well plates and stained with the Annexin V–FITC/PI Apoptosis Detection Kit (KeyGEN BioTECH). Spheroids were monitored in real time; bright-field and fluorescence images were taken using the Cytation 5 MultiMode Microplate Reader (BioTek, USA) every 20 min.

### In vitro DC stimulation assay

BMDCs were generated from the bone marrow of male C57BL/6 mice according to a typical method (*63*). The immature DCs were seeded in a 96-well plate at a density of 8 × 10^4^ cells per well and incubated with C34, NPe6, or CHC in the dark. After 6 hours, the cells were washed and replaced with fresh medium. The cells were further incubated for 24 hours. Then, the DCs were harvested and stained with anti-CD11c–phycoerythrin (PE)–Cy7 (BioLegend, catalog number 117318), anti-CD80–fluorescein isothiocyanate (FITC) (BioLegend, catalog number 104706), and anti-CD86–allophycocyanin (BioLegend, catalog number 105012) antibodies and analyzed by flow cytometry (FCM) (Thermo Fisher Scientific, Attune NxT).

### Detection of ICD in vitro

4T1 cells in the exponential phase were resuspended with serum-free medium at cell densities of 5.4 × 10^5^ cells/ml. C34 was added to DMEM and irradiated by US for 10 min. Following a further incubation of 6 hours, 4T1 cells in each group were harvested and lysed. The extracted total proteins were resolved by gel electrophoresis, which was followed by electroblotting onto a PVDF membrane (Millipore, Billerica, MA, USA). The PVDF membrane was blocked with 5% skim milk in TBST (tris-buffered saline–Tween 20) and immunoblotted with anti-HSP70 antibody and anti-GAPDH (glyceraldehyde phosphate dehydrogenase) antibody (1:1500; Cell Signaling Technology). After further incubation with a horseradish peroxidase–conjugated secondary antibody (1:2000; Univ-Bio), the specific protein bands were visualized with an ECL detection system (Bio-Rad, USA).

The extracellular ATP in conditioned medium that was secreted from treated cells was determined by using an enhanced ATP bioluminescent assay kit (Beyotime Biotechnology, catalog number S0027) according to the manufacturer’s instructions.

### In vivo biodistribution

ICR male mice were used to establish the xenograft H22 tumor model. H22 mouse hepatoma cells (1 × 10^6^ in 50 μl of PBS) were injected subcutaneously into the right side of the mouse back. Mice were intravenously administered C34 at a dose of 16 mg/kg body, and in vivo fluorescence signals of tumors were monitored at different post-injection time points using a small animal imaging system (NightOWL II LB983). Tumor-bearing mice were sacrificed after administration at different time points, and tumor and major tissues were taken for ex vivo HPLC analysis.

C34 was extracted from tissues by a protein precipitation and liquid-liquid extraction method. Methanol was used as the protein-precipitating agent, and chloroform was used to extract C34. HPLC analysis was performed using a Waters 2695 system including Waters 2695 Separations Module and Waters 2489 UV-Vis Detector with an autosampler. Chromatographic separation was performed on a Unitary C18 column (4.6 mm by 250 mm, 5 μm) and maintained at a temperature of 40°C. The mobile phase consisted of 5 mM tetrabutylammonium phosphate monobasic solution (buffer A) and methanol (buffer B). A gradient elution was performed at a flow rate of 1 ml/min, 0 to 10 min, 70 to 95% B; 10 to 15 min, 95 to 100% B; and 15 to 25 min, 100% B. The injection volume was 20 μl, and the detection wavelength was 400 nm.

### Pharmacokinetic study

Sprague-Dawley rats weighing 220 to 250 g were obtained from Liaoning Changsheng Biotechnology (Benxi, China). The animals were fasted overnight with free access to water for at least 12 hours before administration. C34 was intravenously administered at a single dose of 16 mg/kg body weight, and blood samples were collected from the suborbital vein into heparinized tubes at time intervals of 0.083, 0.25, 0.5, 1, 1.5, 2, 4, 6, 12, and 24 hours. Each collected blood sample was immediately centrifuged at 8000 rpm for 10 min. The plasma was separated and frozen below −80°C. The validated method as mentioned above was applied to extraction and HPLC analysis of C34 in plasma. The concentration in plasma at different time points was calculated from the plasma calibration curve. The relevant pharmacokinetic parameters were calculated by a drug and statistics 2.0 program (DAS 2.0, Mathematical Pharmacology Professional Committee of China, Shanghai, China).

### Cross-priming assay in vitro

B16-OVA cells were incubated with NPe6 (25 μM) or C34 (25 μM) and then received irradiation (1.064 MHz, 3.21 W/cm^2^,10 min). iBMDCs were added into each well and incubated for 2 days. The OVAI-specific CD8^+^ T cells were isolated from the splenocytes of OT-1 mice by using a CD8^+^ T cell positive selection kit (STEMCELL) and then added into each well. After incubating for 2 days, all cells were collected and stained for flow cytometry analysis.

### In vivo cytotoxicity assay

Melanoma-bearing mice, which were subcutaneously inoculated with 1 × 10^6^ B16-OVA cells, received C34 + US treatment for two consecutive days. Seven days after the last treatment, splenocytes from naive mice were pulsed with the OVA_257–264_ peptide SIINFEKL and the irrelevant lung carcinoma H-2K^b^ peptide Mut1 FEQNTAQP for 2 hours at 37°C. After washing three times with PBS, OVA_257–264_ peptide-pulsed and Mut1-pulsed splenocytes were incubated with high (4 μM; CFSE^high^) and low (0.5 μM; CFSE^low^) concentrations of carboxyfluorescein diacetate succinimidyl ester (CFSE) (Invitrogen), respectively, for 15 min and inverted them once every 5 min. An equal amount of CFSE^high^- and CFSE^low^-labeled splenocytes was intravenously injected into PBS (control mice)– or C34 + US–treated mice and naive mice as control. Splenocytes were collected after 16 hours, and residual CFSE^high^ and CFSE^low^ target cells remaining in the recipients’ spleens were analyzed by flow cytometry. % Specific cytotoxicity = [1 − (ratio of CFSE^high^/CFSE^low^ obtained from treated mice)/(ratio of CFSE^high^/CFSE^low^ obtained from naive mice)] × 100% (*64*).

### Murine breast cancer model with lung metastases

To establish breast cancer model with spontaneous lung metastasis, 4T1 cells (5 × 10^5^) suspended in PBS were inoculated into the second pair of mammary gland on BALB/c mice. Six days later, the tumors were allowed to reach ~30 mm^3^ before the experiments. Mice were randomly divided into five groups (*n* = 5), including (i) control, (ii) US alone, (iii) C34, (iv) NPe6 + US, and (v) C34 + US. PBS was intravenously injected into the control and US groups. The mice in other groups were intravenously administered with NPe6 or C34 (16 mg/kg). In the US irradiation groups (i.e., US alone, NPe6 + US, and C34 + US), US irradiation (1.064 MHz, 1.88 W/cm^2^, 30 min) was conducted 4 hours after injection. Throughout the experiment, all mice were kept out of the light. The weight and tumor volume of the mice in each group were measured regularly. Three days after the last treatment, the mice were anesthetized with an intraperitoneal injection of Avertin (tribromoethanol), and subcutaneous tumors were extracted and weighed. The in vivo antitumor experiment was repeated four times in this model.

On day 35 after the implantation of 4T1 cells, we performed a PET/CT measurement to evaluate lung metastasis. All scans were performed with a Super Nova small-animal PET/CT scanner (PINGSENG Healthcare, Jiangsu, China). Mice with the orthotopic implantation of 4T1 cells to be monitored by ^18^F-FDG PET/CT were fasted for 24 hours, with access to water only. Body weights were measured, and mice were anesthetized by Avertin and injected using a tail-vein catheter with 10 MBq/0.1 to 0.2 ml of ^18^F-FDG. The lung was scanned by CT. PET data were acquired for 20 min following a delay of 40 min to allow for FDG uptake. In the end, lungs were collected and fixed with Bouin’s solution. Lung micrometastases in five lobes were counted directly through microscopic observation. Serum was collected and analyzed by an enzyme-linked immunosorbent assay (ELISA) kit for IFN-γ. For IFN-γ staining, frozen sections of tumor tissues were fixed with Immunol Staining Fix Solution (Beyotime, catalog number P0098) for 10 min at room temperature, washed three times with PBS, permeabilized with Triton X-100 solution (Beyotime, catalog number P0096) for 20 min, and blocked with Immunol Staining Blocking Buffer (Beyotime, catalog number P0102) for 1 hour. The sections were then incubated with primary antibodies including anti–IFN-γ antibody (1:200 dilution; BioLegend, catalog number 507801) overnight at 4°C. Sections were rinsed, incubated with secondary antibodies (1:200 dilution; ProteinTech, catalog number SA00013-1) in the dark for 1 hour, and then stained with DAPI (4′,6-diamidino-2-phenylindole). The images were acquired using a Leica DMi8 microscope and the THUNDER Imaging System.

### Colon cancer with hepatic metastasis model

Here, we introduced a colorectal cancer hepatic metastasis model using hemiplenectomy with little modification (*65*). BALB/c mice were anesthetized with Avertin (240 mg/kg, intraperitoneally). A left flank incision was made to expose the spleen. The spleen was divided into two hemispleens by using medium-sized Horizon surgical titanium clips, leaving the vascular pedicles intact. With a 27-gauge needle, 3 × 10^5^ viable CT26 cells in 75 μl of PBS were injected into the spleen. Cells then flowed into the splenic and portal veins and were deposited in the liver. The vascular pedicle draining the cancer-contaminated hemispleen was ligated with a small surgical clip. The CT26-contaminated hemispleen was then excised, leaving a functional hemispleen free of tumor cells. The following treatments were the same as the aforementioned procedures. This experiment was repeated two times. At the end of the experiment, mice were sacrificed and livers were excised, weighed, and photographed, and afterward, they were studied by pathological analysis.

### FACS analysis

Tumor tissues, lymph nodes, and spleens were collected from mice after SDT and homogenized into single-cell suspensions following the established protocol (*66*, *67*). Then, the cell suspensions were stained with different antibodies according to the standard protocol. DCs in lymph nodes were stained with anti-CD11c–PE-Cy7 (BioLegend, catalog number 117318), anti-CD80–FITC (BioLegend, catalog number 104706), and anti-CD86–allophycocyanin (BioLegend, catalog number 105012) and analyzed by FCM (Thermo Fisher Scientific, Attune NxT).

To systematically investigate the in vivo antitumor immune responses against tumor metastasis, suspensions in tumors were stained with anti-CD3–PE (BioLegend, catalog number 100308), anti-CD8a–PerCP (BioLegend, catalog number 100732), and anti-CD4–allophycocyanin (BioLegend, catalog number 100412) antibodies. To further analyze T cells in spleens, cells were stained with anti-CD3–PE (BioLegend, catalog number 100308), anti-CD8a–PerCP (peridinin chlorophyll protein) (BioLegend, catalog number 100732), anti-CD4–allophycocyanin (BioLegend, catalog number 100412), anti-CD44–FITC (BioLegend, catalog number 103006), or anti-CD69–FITC (BioLegend, catalog number 104506). All these antibodies used in our experiments were in a 1:100 dilution from the stock solutions.

### Statistical analysis

Statistical analysis was performed by GraphPad Prism 8 using one-way analysis of variance (ANOVA), two-way ANOVA, or Student’s *t* test. The specific statistical test performed for each experiment was included in the corresponding figure legends. Asterisks represent different levels of significance: *P < 0.05, **P < 0.01, ***P < 0.001, and ****P < 0.0001; ns, not significant. All of the flow cytometry analyses were performed using FlowJo 10.

## Notes

View/request a protocol for this paper from *Bio-protocol*.
