# Supplementary material for: An ultrasound-driven immune-boosting molecular machine for systemic tumor suppression
Source: Sci Adv. 2021 Oct 20;7(43):eabj4796. doi: 10.1126/sciadv.abj4796 (PMC8528430; doi:10.1126/sciadv.abj4796)
Supplement: Supplementary file 1 — Supplementary Materials and Methods Figs. S1 to S8 Tables S1 to S3 [file sciadv.abj4796_sm.pdf]

Science Advances
AAAS

Supplementary Materials for

An ultrasound-driven immune-boosting molecular machine for systemic tumor suppression

Liu Wang, Guangzhe Li*, Lei Cao, Yi Dong, Yang Wang, Shisheng Wang, Yueqing Li, Xiuhan Guo, Yi Zhang, Fangfang Sun, Xuemei Du, Jiangan Su, Qing Li, Xiaojun Peng, Kun Shao*, Weijie Zhao*

*Corresponding author. Email: liguangzhe@dlut.edu.cn (G.L.); shaok@dlut.edu.cn (K.S.); zyzhao@dlut.edu.cn (W.Z.).

Published 20 October 2021, *Sci. Adv.* **7**, eabj4796 (2021)
DOI: 10.1126/sciadv.abj4796

This PDF file includes:

Supplementary Materials and Methods
Figs. S1 to S8
Tables S1 to S3

Supplementary Materials and Methods

Synthesis, ^{1}H and ^{13}C NMR and mass spectra data

The general chemicals used in this study were purchased from Energy Chemical Co.. ^{1}H-NMR and ^{13}C-NMR spectra of all compounds were performed with Bruker ADVANCE II 400MHz spectrometer. UV-vis and fluorescence spectra were performed on a Lambda 35 UV-visible spectrophotometer (PerkinElmer) and VAEIAN CARY Eclipse fluorescence spectrophotometer (Serial No. FL0812-M018), respectively. Mass spectrometric (MS) data were detected by using LTQ Orbitrap XL instruments.

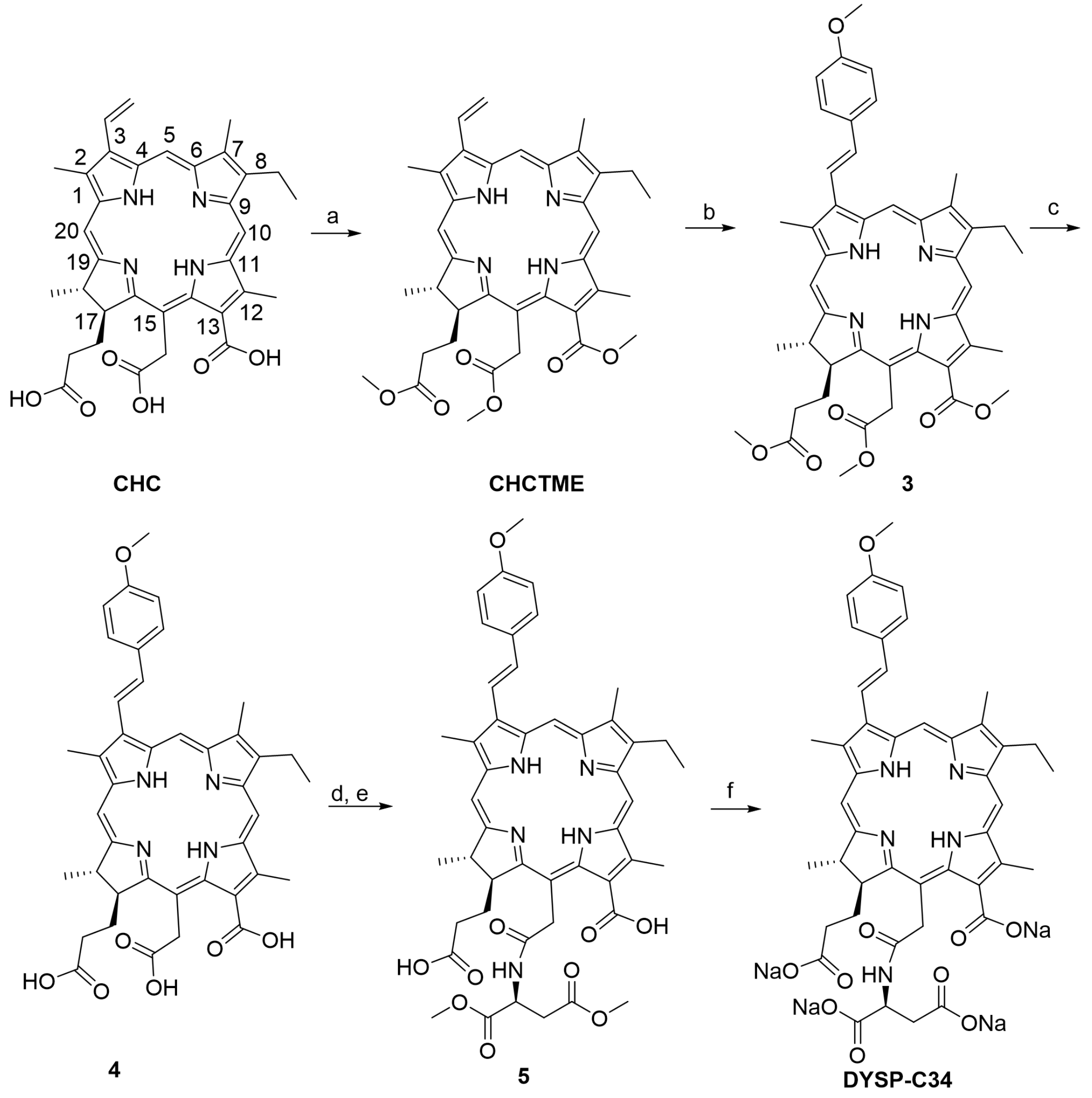

Fig. S1. The synthetic routes of DYSP-C34. Reagents and reaction conditions: (a) CH_3I, K_2CO_3, DMF, RT, 10 h, 93%; (b) 4-Methoxystyrene, Grubbs' Catalyst, CH_2Cl_2, 40°C, reflux, 24 h, 55%; (c) 1M KOH, THF, 45°C, reflux, 12 h; (d) EDCI, DMF, 35°C, 2 h; (e), L-Asp $(OMe)_2$ HCl, Et_3N, 35°C, 2 h; (f) 1M NaOH, acetone, 45°C, reflux, 12 h, 51%.

Chlorophyll a degradation product CHC (Chenghai Chlorin, the same as Chlorin e6) was prepared from Spirulina platensis in Yunnan Chenghai Lake of China. To expand conjugation system and alter the charge distribution of chlorin macrocycle. The aromatic group was conjugated at C-3^2 through Ru catalyzed olefin metathesis with CHCTME (CHC trimethyl ester). The aspartic acid side chain was introduced at C-15^2 regio-selectively using EDCI as condensing agent, followed hydrolysis under NaOH condition gave target **DYSP-C34** as sodium salts, which possessed desired long-wavelength absorption and water solubility simultaneously.

Synthesis of CHC trimethyl ester (**CHCTME**)

To a stirred solution of **CHC** (1.02 g) in DMF (11 mL) was added K_2CO_3 (4.63 g) and CH_3I (1 mL), the mixture was stirred for 10 h at room temperature under N_2 atmosphere. The residues were diluted with EA (100 mL) and washed with water (3 x 100 mL) and NaCl saturated aqueous solution (100 ml). The organic layer was dried over anhydrous Na_2SO_4 and evaporated. The residue was purified by silica gel column chromatography (Petroleum ether: Acetone = 6: 1) to afford **CHCTME** (1.0 g, yield 93%) as a dark green solid. ^{1}H-NMR (400 MHz, $CDCl_3$)**:** δ 9.7 (1H, H-5, s), 9.5 (1H, H-10, s), 8.7 (1H, H-20, s), 8.0 (1H, H-3^1, dd, J = 11.5, 17.8 Hz), 6.3 (1H, H-3^{2a}, dd, J = 1.4, 17.8 Hz), 6.1 (1H, H-3^{2b}, dd, J = 1.4, 11.5 Hz), 5.4 (1H, H-15^{1a}, d, J = 18.8 Hz), 5.2 (1H, H-15^{1b}, d, J = 18.8 Hz), 4.4 (1H, H-18, q, J = 7.2 Hz), 4.4 (1H, H-17, dd, J = 1.9, 10.3 Hz), 4.3 (3H, -CH_3, s), 3.7 (2H, H-8^1, q, J = 7.6 Hz), 3.8 (3H, -CH_3, s), 3.6 (3H, -CH_3, s), 3.6 (3H, -CH_3, s), 3.4 (3H, -CH_3, s), 3.3 (3H, -CH_3, s), 2.5 (1H, H-17^{1a}, m), 2.2 (1H, H-17^{2a}, m), 2.2 (1H, H-17^{1b}, m), 1.8 (1H, H-17^{2b}, m), 1.7 (3H, H-18^1, d, J = 7.2 Hz), 1.7 (3H, H-8^2, t, J = 7.6 Hz), -1.3 (1H, N-H, s), -1.5 (1H, N-H, s); ^{13}C-NMR (101 MHz, $CDCl_3$): δ 173.6, 173.0, 169.6, 169.5, 166.9, 154.8, 148.9, 145.0, 139.5, 136.4, 135.9, 135.4, 135.4, 134.8, 130.5, 129.4, 128.7, 123.4, 121.7, 102.2, 102.1, 98.7, 93.6, 77.2, 53.0, 52.1, 51.6, 49.4, 38.6, 31.1, 29.6, 22.9, 19.7, 17.7, 12.4, 12.1, 11.3; HRMS (ESI) m/z Calcd for $C_{37}H_{43}N_4O_6$ $[M+H]^+$: 639.3183, Found: 639.3173.

Synthesis of 3^2-(4-methoxyphenyl)-CHC (**3**)

A solution of **CHCTME** (1.0 g), 4-Methoxystyrene (751.9 mg) and Grubbs' Catalyst (398.6 mg) in anhydrous CH_2Cl_2 (52 mL) was stirred for 24 h at 40°C under N_2 atmosphere. The mixture was diluted with CH_2Cl_2 (50 mL) then washed with saturated NH4Cl solution (100 mL) and saturated NaCl solution (100 mL) respectively. The organic phase was dried over anhydrous Na_2SO_4, and evaporated under reduced pressure. The residue was purified by silica gel column chromatography (Petroleum ether: Acetone = 8: 1) to give compound **3** (0.64 g, yield 55%) as a brownish black solid. ^{1}H-NMR (400 MHz, $CDCl_3$): δ 9.7 (s, 1H), 9.6 (s, 1H), 8.8 (s, 1H), 8.3 (d, J = 16.4 Hz, 1H), 7.8 (d, J = 8.6 Hz, 2H),7.7 (d, J = 16.4 Hz, 1H), 7.1 (d, J = 8.6 Hz, 2H), 5.4 (d, J = 18.8 Hz, 1H), 5.3 (d, J = 18.8Hz, 1H), 4.5 (m, 2H), 4.3 (s, 3H), 4.0 (s, 3H), 3.8 (s, 3H), 3.8 (q, J = 7.7 Hz, 2H), 3.7 (s,3H), 3.6 (s, 3H), 3.5 (s, 3H), 3.3 (s, 3H), 2.6 (m, 1H), 2.3 (m, 2H), 1.8 (m, 1H), 1.8 (d, J = 7.2 Hz, 1H), 1.7 (t, J = 7.7 Hz, 3H)， -1.2 (s, 1H), -1.3 (s, 1H); HRMS (ESI) m/z Calcd for $C_{44}H_{49}N_4O_7$ $[M+H]^+$: 745.3601, Found: 745.3633.

Synthesis of 3^2-(4-methoxyphenyl)-CHC (4) and 3^2-(4-methoxyphenyl)-15^2- Asp $(OMe)_2$-CHC (**5**)

To a solution of compound **3** (650 mg) in THF (40 ml) was added 1 M KOH aqueous solution (40 ml). The mixture was stirred for 10 h at 50°C under N_2 atmosphere and then concentrated under vacuum to remove THF. The pH of the resulting water phase was adjusted to 2 with 2 M

HCl aqueous solution and the precipitates was filtered using a Buchner funnel. The residue was washed with 1% propionic acid aqueous solution and dried under vacuum at 30°C to give crude compound **4**, which was used directly in the next step.

To the above compound **4** (650 mg) in dried DMF (9 mL) was added EDCI (212.9 mg), the mixture was stirred for 1 h at 35°C under N_2 atmosphere. To the above mixture was then added Et_3N (452 µl) and L-Asp(OMe)$_2$·HCl (367.6 mg) and the resulting solution was stirred for additional 1 h at 35°C. The mixture was poured into 1% HCOOH aqueous solution (200 mL) and the precipitates was filtered. The residue was washed with 1% propionic acid aqueous solution and further purified by silica gel column chromatography ($CHCl_3$/MeOH/H_2O = 60:3:1) to give compound **5** (620 mg) as a brownish black solid. ^{1}H NMR (400 MHz, Acetone): δ 9.8 (s, 1H), 9.7 (s, 1H), 9.05 (s, 1H), 8.4 (d, J = 16.6 Hz, 1H), 7.8 (d, J = 8.2 Hz, 2H), 7.7 (d, J = 16.6 Hz, 1H), 7.1 (d, J = 8.2 Hz, 2H), 5.4 (m, 2H), 4.7 (s, 1H), 4.7 (d, 1H), 4.6 (d, 1H), 3.9 (s, 3H), 3.8 (m, 2H), 3.6 (s, 3H), 3.5 (s, 3H), 3.5 (s, 3H), 3.4 (s, 3H), 3.3 (s, 3H), 2.96 (m, 2H), 2.8 (d, 2H), 2.4 (m, 2H), 2.9(m, 1H), 1.8 (d, 3H), 1.7 (m, 4H); HRMS (ESI) m/z Calcd for $C_{47}H_{52}N_5O_{10}$ $[M+H]^+$: 846.3714, Found: 846.3713.

<u>Synthesis of 3^2-(4-methoxyphenyl)-15^2- Aspartyl-CHC (**DYSP-C34**)</u>

To a solution of compound **5** (620 mg) in acetone (15 ml) was added 1 M NaOH aqueous solution (15 ml). The mixture was stirred for 10 h at 45°C under N_2 atmosphere. Absolute ethyl alcohol (150 ml) was slowly added to the above mixture and the precipitates was filtered. The residue was dissolved in deionized water and evaporated under reduced pressure. The residue was recrystallized from absolute ethyl alcohol and deionized water to give **DYSP-C34** (400 mg, yield 55% over 3 steps) as a red brown solid. ^{1}H-NMR (D_2O, 400 MHz, ppm): δ 9.4(s,1H,10-H), 9.0(s,1H,5-H), 8.2(s,1H,20-H), 6.3(d, 1H, J=17.9, 3^1-H), 5.9(d, 1H, J=16.2, 15^{1a}-H), 5.8(d, 1H ,J=17.9, 32-H), 5.5(m, 4H, 2', 3', 5', 6'-H), 5.3(d, 1H, J=16.2, 15^{1b}-H) , 5.1 (t, 1H, 15Asp-2'-H), 5.1(m, 1H, 17-H), 5.1(m, 1H, 18-H), 3.8 (s, 3H, 12^1-H), 3.3 (s, 3H, 4'-H), 3.3(s, 2H, 8^1-H), 3.2 (m, 1H, 17^{2a}-H), 3.0(m, 1H, 17^{2b}-H), 2.9 (m, 2H, 15-Asp-1'-H), 2.9(m, 3H, 17^{1a}-H), 2.4 (s, 3H, 7^1-H), 2.4(m, 1H, 17^{1b}-H), 2.3(m, 4H, 2^1-H), 2.2 (d, 3H, 18^1-H), 1.3(t, 3H, 8^2-H); ^{13}C-NMR (400 MHz, D_2O): δ 182.9(Asp-C=O), 179.6 (Asp-C=O), 178.8 (17^3-C), 177.4 (15^2-C), 175.0(13^1-C), 170.0(19-C), 164.6 (16-C), 158.0(ph1-C), 152.6 (6-C), 149.2(9-C), 144.4(8-C), 138.4(1-C), 135.8 (7-C), 135.7 (11-C), 134.3(14-C), 133.9(4-C), 133.7(3-C), 133.1(3^1-C), 131.3(2-C), 129.7(ph-3-C), 128.6 (12-C), 126.5 (ph-4-C), 117.3(3^2-C), 113.3(ph-5-C), 102.9(15-C), 100.7(10-C), 98.4(5-C), 94.3(20-C), 55.2(ph-4'C), 54.4(asp-αC), 54.2(17-C), 49.6(18-C), 41.7(asp-$^\beta$C), 40.0(15^1-C), 36.4(17^{1a}-C), 32.7(17^{1b}-C), 23.9(18^1-C), 18.9(8^1-C), 16.9(8^2-C), 11.8(12^1-C), 11.0(2^1-C), 10.4(7^1-C); HRMS (ESI) m/z Calcd for $C_{45}H_{46}N_5O_{10}$ $[M-4Na+3H]^-$: 816.3245, Found: 816.3256.

Copies of ^{1}H-NMR, ^{13}C-NMR spectra

CHCTME ^{1}H-NMR (400 MHz, $CDCl_3$)

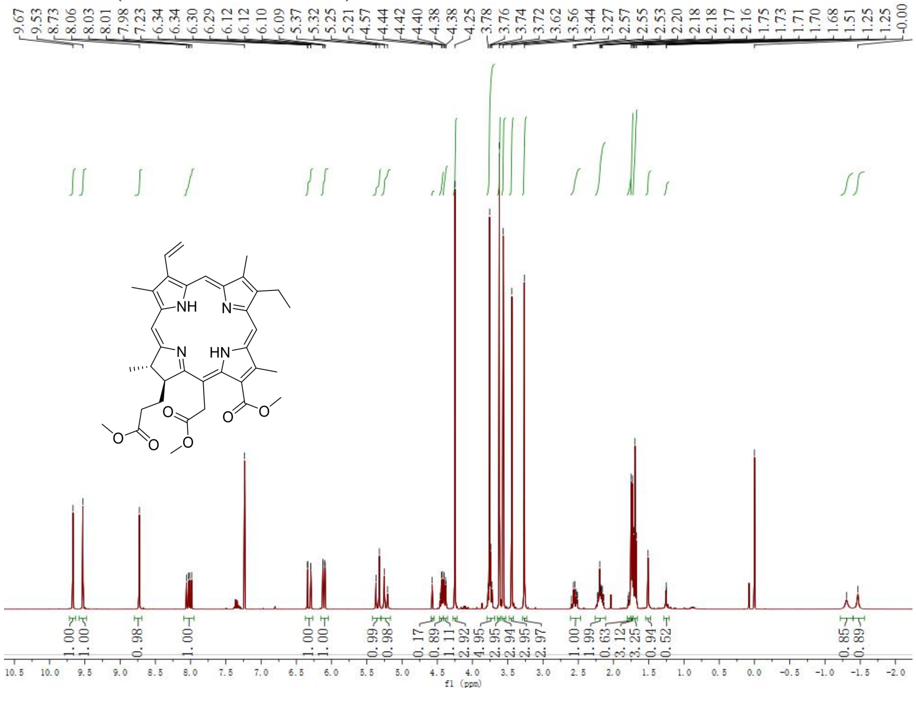

CHCTME ^{13}C-NMR (400 MHz, $CDCl_3$)

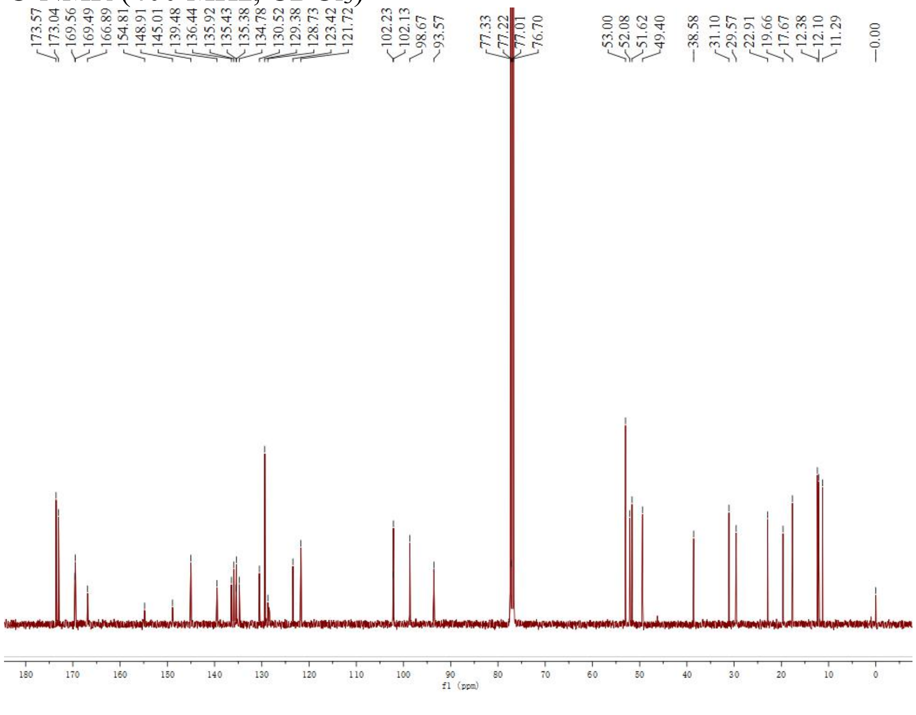

Compound 3 ^{1}H-NMR (400 MHz, $CDCl_3$)

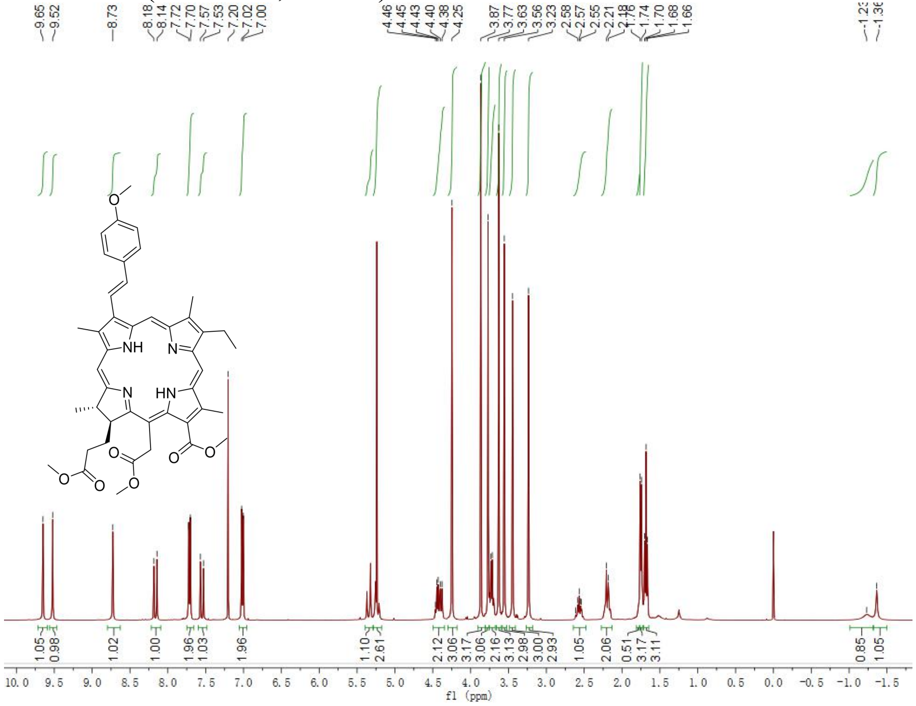

Compound 5 ^{1}H-NMR (400 MHz, Acetone)

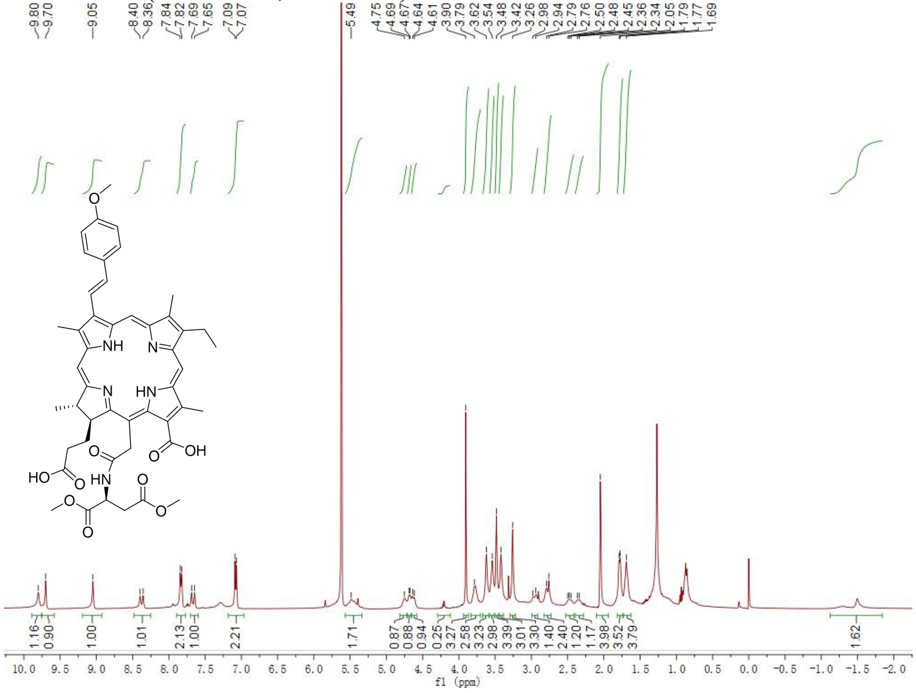

DYSP-C34 ^{1}H-NMR (400 MHz, D_2O)

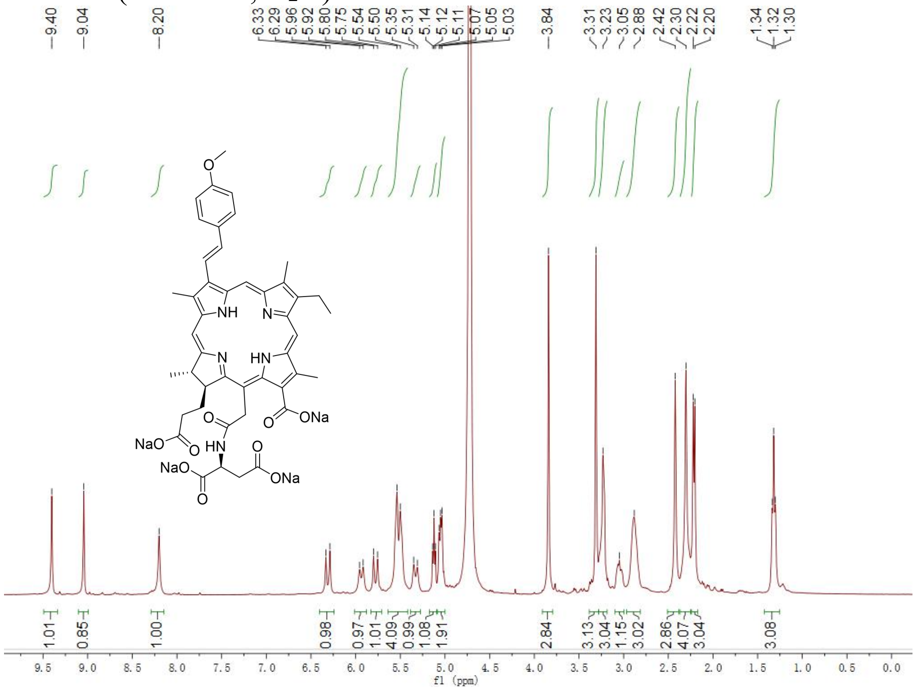

DYSP-C34 ^{13}C NMR (400 MHz, D_2O)

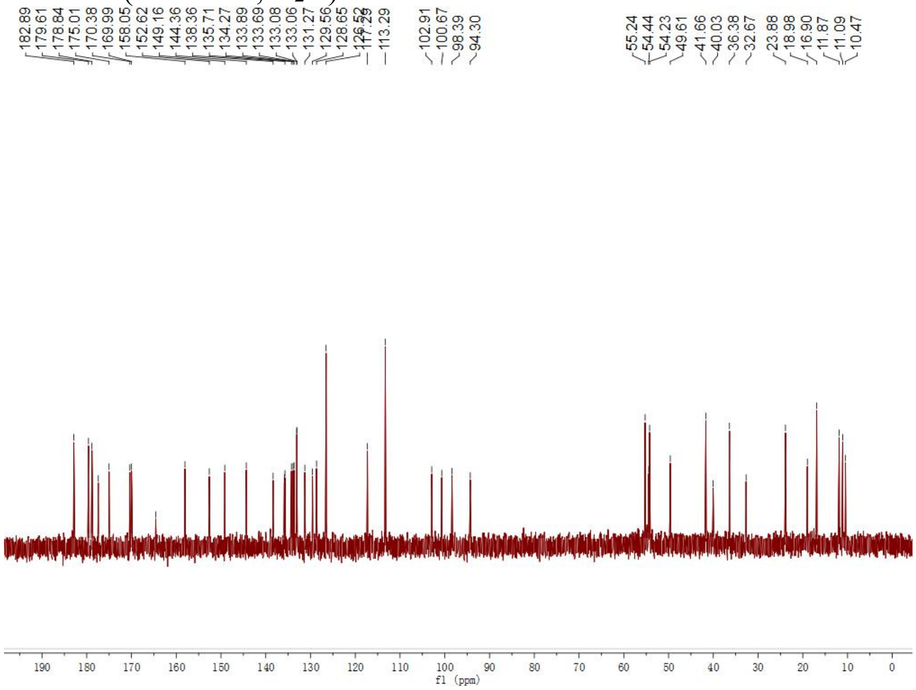

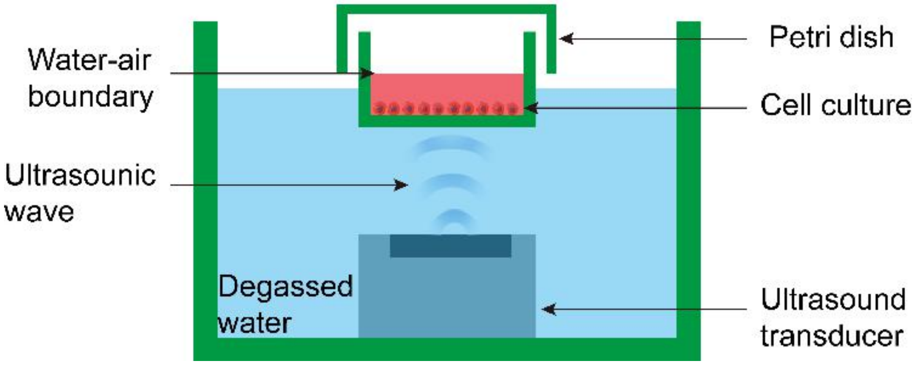

Fig. S2. Typical ultrasound exposure setup for *in vitro* study.

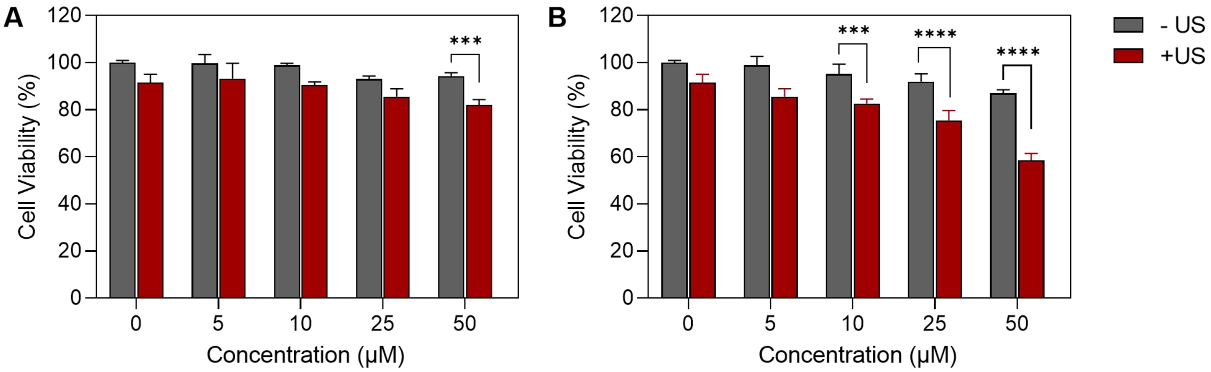

Fig. S3. **SDT efficiency comparison of NPe6 and C34 *in vitro*.** Cell viabilities of MCF-7 cells after co-incubation with (**A**) NPe6 or (**B**) C34 at varied concentrations for 0.5 h and exposed to US irradiation (1.0 MHz, 3.21 W/cm^2). Data were presented as mean ± SD (n=3) and were compared using two-way ANOVA, ***$p < 0.001$, ****$p < 0.0001$.

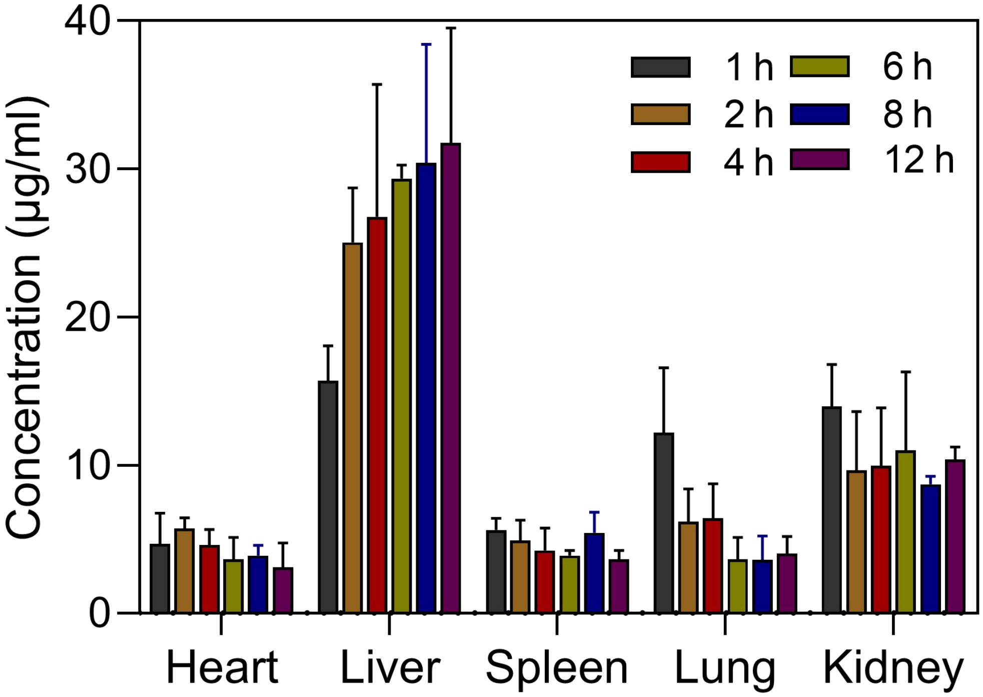

Fig. S4. Concentration-time profiles of C34 in tumor-bearing mice tissues. The mice were *i.v.* administered of C34 at a dose of 16 mg/kg body wight. Data were presented as mean ± SD (n = 5 to 6).

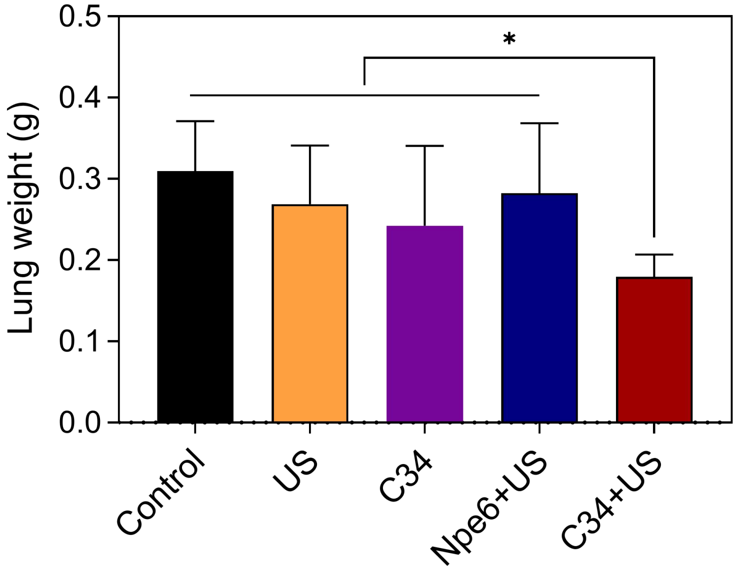

Fig. S5. Average weights of excised lungs at the end of treatments. Values were means ± SD (n = 4). Data were presented as mean ± SD (n = 4). P values were calculated using one-way ANOVA with Dunnett's multiple comparison test, $*p < 0.05$.

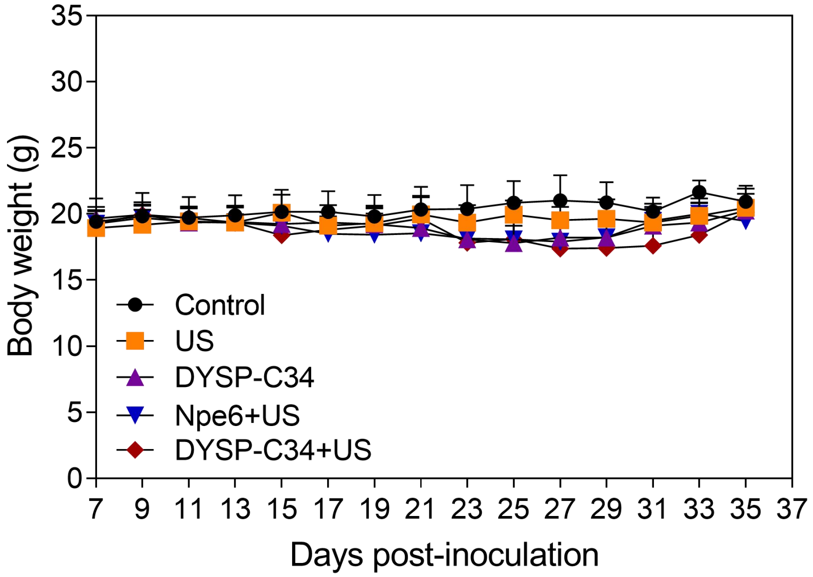

Fig. S6. Body weight curves versus the number of days post different treatments. Data were presented as mean ± SD ($n \geq 5$).

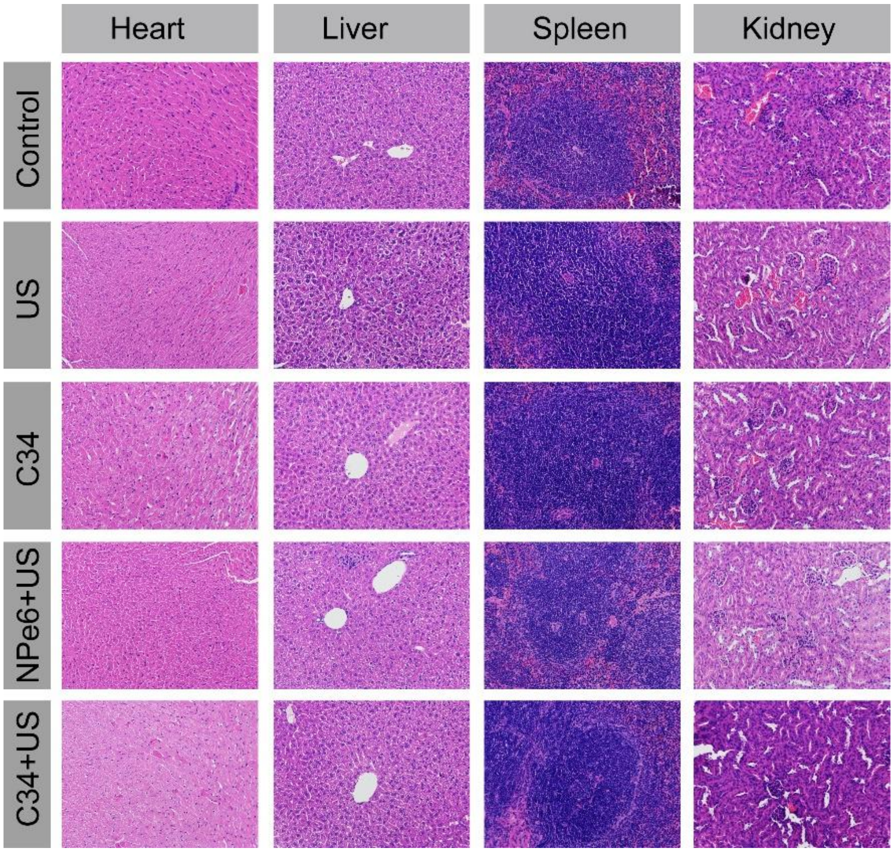

Fig. S7. H&E-staining for safety evaluation. Tissue sections of major organs (heart, liver, spleen and kidney) from tumor-bearing mice after different treatments.

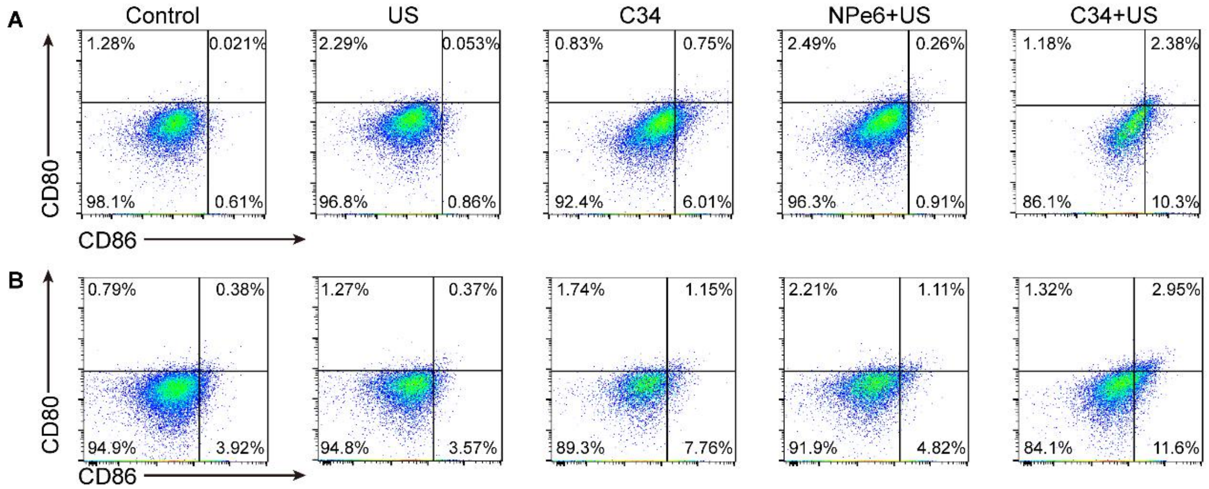

Fig. S8. Representative flow-cytometry plots of DCs maturation. DCs maturation in the (**A**) tumor tissues and (**B**) tumor-draining lymph nodes induced by C34-mediated SDT, as assessed by flow cytometry after staining with CD11c, CD80 and CD86.

Table S1. p_{RMS} measured with needle hydrophone under free-field ultrasound condition.

P_A (W)	I_{SATA} (W/cm^2)	p_{RMS} /Mpa
3.0	0.91	0.057
6.2	1.88	0.095
10.6	3.21	0.117

Table S2. Calibration curves and correlation coefficients (r^2) of C34 in plasma and tissue samples from mice.

Sample	Calibration curve	r^2
Plasma	y = 0.3170x - 1.021	0.9986
Heart	y = 0.1524x - 0.1129	0.9969
Liver	y = 0.1274x - 0.0817	0.9967
Spleen	y = 0.1760x - 0.1571	0.9966
Lung	y = 0.1697x + 0.034	0.9915
Kidney	y = 0.1260x - 0.1792	0.9919
Tumor	y = 0.2716x - 0.1717	0.9979

y: peak area ratio (C34/IS); x: added C34 concentration (µg/ml).

Table S3. The main pharmacokinetic parameters of C34 in rat plasma (n = 5).

Non-compartmental mode was used to calculate the pharmacokinetic parameters with DAS 2.0, and the studied parameters include area under the plasma concentration curve (AUC), mean residence time (MRT), half-life ($t_{1/2}$), clearance (CL) and apparent volume of distribution (V).

Parameters	Unit	Value
$AUC_{(0-t)}$	mg/L/h	934.51 ± 59.389
$AUC_{(0-\infty)}$	mg/L/h	1025.008 ± 62.329
$MRT_{(0-t)}$	h	6.772 ± 0.379
$MRT_{(0-\infty)}$	h	9.191 ± 0.976
$t_{1/2z}$	h	6.984 ± 0.646
$CL_{Z/F}$	L/h/kg	0.016 ± 0.001
$V_{Z/F}$	L/kg	0.158 ± 0.017
